# Genome-wide association analysis identifies seven loci conferring resistance to multiple wheat foliar diseases, including brown and yellow rust resistance originating from *Aegilops ventricosa*

**DOI:** 10.1007/s00122-025-04907-x

**Published:** 2025-06-02

**Authors:** Keith A. Gardner, Bethany Love, Pauline Bansept-Basler, Tobias Barber, Simon Berry, Nick Bird, Finn Borum, Lesley Boyd, James K. M. Brown, Ruth Bryant, Paul Fenwick, David Feuerhelm, Nick Gosman, Charlotte Hayes, Tina Henriksson, Peter Jack, Rachel Kirby, Matt Kerton, Jacob Lage, Linda Kærgaard Nielsen, Gemma Rose, Rajiv Sharma, Jörg Schondelmaier, Stephen Smith, Pernilla Vallenback, Duncan Warner, The WAGTAIL Consortium, Tally I. C. Wright, Camila M. Zanella, James Cockram, Ian J. Mackay, Donal M. O’Sullivan

**Affiliations:** 1 NIAB, 93 Lawrence Weaver Road, Cambridge, CB3 0LE UK; 2 Limagrain UK Ltd, Market Rasen, Lincolnshire, LN7 6DT UK; 3 KWS UK Ltd, 56 Church Street, Thriplow, Hertfordshire, SG8 7RE UK; 4 Sejet Planteforædling, 8700 Horsens, Denmark; 5 John Innes Centre, Norwich Research Park, Norwich, NR4 7UH UK; 6 RAGT Seeds Ltd, Grange Road, Ickleton, Saffron Walden, CB10 1TA UK; 7 Syngenta, Hill Farm Road, Whittlesford, Cambridge, CB22 4QT UK; 8 Elsoms Wheat Ltd, Pinchbeck Road, Spalding, Lincolnshire, PE11 1QG UK; 9 Lantmännen Lantbruk, Svalöv, Sweden; 10 DSV UK Ltd, Wardington Road, Wardington, Banbury, Oxfordshire OX17 1FE UK; 11 Scotland’s Rural College (SRUC), West Mains Road, Edinburgh, EH9 3JG UK; 12 Saaten-Union Biotec GmbH, Hovedisser Str. 94, 33818 Leopoldshoehe, Nordrhein-Westfalen Germany; 13 University of Reading, Reading, RG6 6EU UK

## Abstract

We assembled a European bread wheat (*Triticum aestivum* L.) association mapping panel (*n* = 480) genotyped using a 90,000 feature single nucleotide polymorphism array, with the aim of identifying genetic loci controlling resistance to four fungal diseases: yellow (stripe) rust (YR), brown (leaf) rust (BR), Septoria tritici blotch (ST) and powdery mildew (PM). Simulations showed our panel to have good power to detect genetic loci, with > 50% probability of identifying loci controlling as little as 5% of the variance when heritability was 0.6 or more. Using disease infection data collected across 31 trials undertaken in five European countries, genome-wide association studies (GWAS) identified 34 replicated genetic loci (20 for YR, 12 for BR, two for PM, 0 for ST), with seven loci associated with resistance to two or more diseases. Construction and analysis of eight bi-parental populations enabled two selected genetic loci, yellow rust resistance locus YR_2A010 (chromosome 2A) and YR_6A610 (6A), to be independently cross-validated, along with the development of genetic markers to track resistance alleles at these loci. Notably, the chromosome 2A yellow and brown rust resistance locus corresponds to the 2N^v^S introgression from the wild wheat species, *Aegilops ventricosa*. We found evidence of strong selection for 2N^v^S over recent breeding history, being present in 48% of the most recent cultivars in our panel. Collectively, we define the genetic architectures controlling resistance to major fungal diseases of wheat under European field environments, and provide resources to exploit these for the development of new wheat cultivars with improved disease resistance.

**Supplementary Information:**

The online version contains supplementary material available at 10.1007/s00122-025-04907-x.

## Introduction

Diseases of wheat (*Triticum aestivum* L.) can have significant impact on grain quality and yield, with an estimated potential yield loss of 20% per year (Wulff and Krattinger 2022). Accordingly, growers employ various methods to help prevent or control disease in their crops. Ideally, integrated pest management approaches are applied, which combine action thresholds with disease monitoring, prevention and control measures. Disease prevention via growth of cultivars with good genetic resistance is a key component of such strategies. This is particularly true in situations where the cost of fungicides and pesticides are restricting factors, or where regulations restrict the use of specific chemical control options. Indeed, legislative regulation is likely to become increasingly focused on encouraging sustainable agricultural approaches, therefore promoting efficient exploitation of genetic sources of crop resistance. For example, the recent Farm to Fork Strategy, a central component of the European Green Deal, aims to encourage food systems that are fair, healthy and environmentally friendly (European Commission communication COM (2020) 381 final).

### Target wheat diseases: yellow rust, brown rust, powdery mildew and Septoria tritici blotch

In north-western Europe, four of the most damaging fungal diseases of wheat are yellow rust (YR, also known as stripe rust; caused by *Puccinia striiformis* Westend f. sp. *tritici*, hereafter termed *Pst*), brown rust (BR, also known as leaf rust; caused by *Puccinia triticina* Erikss., *Pt*), Setporia tritici blotch (caused by *Zymoseptoria tritici*, *Zt*) and powdery mildew (caused by *Blumeria graminis* f. sp. *tritici*, *Bgt*). *Pst*, *Pt* and *Bgt* are obligate biotrophic fungi that require living host tissue to complete their lifecycle. In contrast, *Zt* has been classified as a latent necrotroph, initially growing asymptomatically in host tissue after which a necrotrophic phase is initiated during which host cell death is rapidly induced (Sanchez-Vallet et al. 2015). All four diseases predominantly result in infection of wheat leaves and can result in notable reductions in grain yield and quality if left unchecked. The causal agents of yellow rust and brown rust belong to the same fungal genus and have complicated lifecycles involving numerous spore stages as well as multiple plant host species for completion of their lifecycles (reviewed by Bouvet et al. 2022a; Ren et al. 2023). Their asexual stages are undertaken on wheat, with infection initiated via wind-blown spores (termed urediniospores for *Pst*, and urediospores for *Pt*), resulting in the development of pustules on the surfaces of infected wheat leaves that release spores, which can reinfect wheat plants, so continuing the asexual lifecycle phase. For yellow rust, the yellow or orange pustules are arranged in stripes along the leaf blade, while brown rust pustules are brown and are arranged without specific pattern. Although brown rust tends to develop later in the season than yellow rust, both diseases lead to loss of green leaf area, thus affecting yield. Powdery mildew is characterised by pale pink asexual colonies on the surfaces of infected wheat leaves, with infection most prominent in years with mild temperatures and high humidity. Release of conidia from these colonies can lead to reinfection cycles as quick as five days (Rana et al. 2022). Septoria tritici blotch results from the infection of a hemi-trophic fungus. Thus, while *Zt* initially requires living host tissue for infection, the fungus subsequently kills and takes up nutrients from the dead host tissues (Gupta et al. 2023). The visual symptoms of Septoria tritici blotch include elongated chlorotic or necrotic lesions on the leaves, which because they are restricted by the leaf veins, are typified by rectangular appearance. Within-season spread of Septoria tritici blotch infection is typically mediated via rain splash spread of pycnidiospores asexually produced from the characteristic small black fruiting bodies (pycnidia) that form on infected areas.

### Wheat genetic resistance to target fungal diseases

Wheat genetic resistance to fungal infection is typically classified as either all-stage resistance (also termed ‘race-specific resistance’ or ‘seedling resistance’) or adult plant resistance (‘race nonspecific resistance’). All-stage resistance is expressed at the seedling stage and extends throughout plant development. It is underpinned by the gene-for-gene model (Flor 1956), with the underlying genes in wheat typically encoding nucleotide-binding site, leucine-rice repeat (NBS-LRR) proteins. Use of cultivars with low numbers of all-stage resistance genes over large areas of cultivation can result in high pathogen selection pressures, leading to the evolution of pathogen races able to overcome specific sources of all-stage resistance—presumably via mutation or deletion of the pathogen effector proteins that specific NBS-LRR proteins detect. In Europe, a recent example is the breakdown of the yellow rust resistance conferred by *Yr17*, resulting in growers rapidly shifting to cultivars that carried other sources of resistance (Bayles et al. 2000). Such cycles of ‘boom and bust’ can be ameliorated by the use of cultivars that pyramid multiple all-stage resistance loci and carry sources of adult plant resistance. Indeed, adult plant resistance loci typically provide more durable resistance, which, while quantitative in nature, are less prone to being overcome by fungal pathogens. Some sources of adult plant resistance confer resistance to multiple fungal pathogens. For example, resistance to yellow rust, leaf rust (*Lr*), stem rust (*Sr*) and powdery mildew (*Pm*) is conferred by the same resistance gene *Yr18/Lr34/Sr67Pm38* and has often been used in cultivars developed via the CIMMYT international breeding programme (Singh et al. 2005). While relatively few adult plant resistance genes have been cloned, they do not belong to a single class of gene: *Yr36* encodes a protein with a kinase and a START lipid-binding domain (Fu et al. 2009), *Yr18/Lr34* encodes an ABC transporter (Krattinger et al. 2009) and *Yr46/Lr67* encodes a hexose transporter (Moore et al. 2015). Additionally, some wheat genes play an essential role in pathogen colonisation and their mutation/deletion can result in increased resistance. Examples include *mildew resistance locus* (*Mlo*) (Buschges et al. 1997), a branched-chain amino acid aminotransferase termed *TaBCAT1* (Corredor-Moreno et al. 2021) and a cytoplasmic protein kinase termed *TaPsIPK1* (Wang et al. 2022). The majority of adult plant resistance, however, is conferred by genes with small individual effects but which collectively provide effective disease control. For further details of the genes and genetics of wheat resistance to the four fungal pathogens investigated here, see Bouvet et al. (2022a) (yellow rust), Ren et al. (2023) (brown rust), Bapela et al. (2023) (powdery mildew) and Ababa (2023) (Septoria tritici blotch).

### Safeguarding future wheat production: understanding the genetics of resistance in current cultivars

Knowledge of which disease resistance loci are deployed in current wheat cultivars helps inform resistance breeding strategies. For many cloned genes, molecular markers are now available that allow resistance loci to be tracked within breeding programmes (e.g. Rasheed et al. 2016). However, systematic understanding of the full repertoire of resistance loci deployed within elite wheat genepool will provide a framework from which informed resistance breeding can be conducted and helps safeguard against the sudden collapse of genetic resistance in contemporary cultivars. For example, sources of yellow rust adult plant resistance identified in a multi-founder wheat population have been shown to be rare in north-west European germplasm (Bouvet et al. 2022b, 2022c), indicating that their wider deployment in new cultivars could aid resistance durability. In the European context, additional factors such as the rapid change in genetic diversity and virulence of the yellow rust fungus *Pst* since the year 2000 (Hovmøller et al. 2007) which from 2011 began to largely replace the previously clonal *Pst* isolates (Hovmøller et al. 2016; Hubbard et al. 2015), and the relatively low number of assayed resistance loci conferring brown rust resistance in current surveys of UK *Pt* isolates (UKCPVS 2022) further highlight the need to optimise understanding and deployment of sources of wheat genetic resistance. Moreover, although powdery mildew resistance is relatively high in UK wheat and Septoria tritici resistance has increased over the past 30 years, little is known the genetic structure of resistance to either disease (Brown 2021). With current winter wheat UK Recommended List varieties averaging resistance rating scores of around 6 for powdery mildew and Septoria tritici blotch (on a 1–9 non-linear scale, where 1 = susceptible. AHDB, 2024), there is of course scope for further genetic improvement despite the successes of the past.

Genome-wide association studies (GWAS) allow the genetic architecture of target traits to be undertaken in large collections of contemporary germplasm (e.g. Mellers et al. 2020) and can offer superior mapping precision compared to conventional segregating populations (Gardiner et al. 2020). Here, we assembled an association mapping panel of 480 predominantly European winter wheat cultivars released between 1916 and 2007 and genotyped using a 90,000 feature single nucleotide polymorphism (SNP) array. We then assessed the panel for resistance to four fungal diseases—yellow rust, brown rust, powdery mildew and Septoria tritici blotch—via 31 field trials across five European countries, allowing identification of resistance loci by GWAS. Finally, we selected two genetic loci for independent validation in eight bi-parental populations and provided genetic markers for further investigation and molecular tracking of the loci.

## Methods

### Association mapping panel and genotyping

A panel of 480 mainly winter wheat cultivars and breeding lines that represent the north-western European wheat elite breeding genepool of recent decades was assembled from previous germplasm collections and participating breeding companies (Supplementary Table S1). For each accession, a single seed was grown, genomic DNA extracted (Fulton et al. 1995), and self-fertilised seed produced for downstream research. Genotyping was performed using a wheat Illumina iSelect 90,000 feature SNP array (Wang et al. 2014), with genotypes called with GenomeStudio (Illumina). All genotypes scored as 0 (A:A) or 1 (B:B), with the very rare cases of heterozygotes (A:B), were treated as “NA”. The resulting genotypic dataset was processed to remove markers with missing data ≥ 10%, before the remaining missing values in the genotypic data were imputed using the R package missForest (Stekhoven and Buehlmann 2012) with 200 trees. Markers with a minor allele frequency ≤ 2.5% were then removed in the imputed dataset.

### Forming a pseudo-genetic map

The 90 k marker probe DNA sequences (Wang et al. 2014) were used as queries against the wheat reference genome of cultivar Chinese Spring (RefSeq v1.0; IWGSC 2018) via BLAST + 2.7.1 using default parameters (Camacho et al. 2009). For each hit, the median base pair between the start and stop locations were taken as the physical position of the marker. The MAGIC 90 k genetic linkage map from Gardner et al. (2016) was used to aid the marker anchoring to physical genome locations. Using R (R Core Team 2020), three steps were applied to anchor markers: (1) If a marker had a singular physical hit for the same chromosome mapped in the genetic linkage map, that hit was taken as the anchored position. (2) For each marker not anchored in the first step, pairwise correlation (*r*^2^) was calculated with all markers already anchored to find the pair that yielded the highest *r*^2^. If the *r*^2^ value was above a determined threshold (*r*^2^ > 0.35) and the unanchored marker had at least one physical hit on the same chromosome as the anchored marker, then the closest physical hit to the anchored marker was taken as the anchored position. (3) A backwards control step was implemented where every marker (*m*_1_) was correlated with the next two markers along the chromosome (*m*_2_ and *m*_3_). If *r*^2^ between *m*_1_ and *m*_3_ was > 0.7, *r*^2^ between *m*_1_ and *m*_2_ was < 0.35 and *r*^2^ between *m*_2_ and *m*_3_ was < 0.35, then *m*_2_ was excluded from the anchored markers. Finally, the R package LDheatmap (Shin et al. 2006) was used to inspect the resulting linkage disequilibrium (LD) between the final 20,166 anchored markers. These markers, along with the 5366 unanchored SNPs, were then ‘skimmed’ to remove markers that were 100% correlated to each other, using a custom R script. The skimming approach involved removing a marker in each pair of markers with an absolute correlation coefficient (*r*) = 1. This resulted in 11,858 markers (8962 anchored SNPs and 2896 unmapped SNPs). All genotypic data are available online at www.niab.com/resources/.

### Field trials, phenotypic data and trial analysis

Phenotypic data were collected from 31 autumn-sown field trials (Table 1), grown in the UK (21 trials), Germany (4), Denmark (4), France (1) and Sweden (1) over four years (harvest years 2012, 2013, 2014, and 2015). For all but one trial (ST_4), two replicate plots for each entry were grown per trial, with inclusion of susceptible control cultivars at higher replicate number. Entries were randomised between two main blocks, typically with inclusion of additional sub-blocks. Further details of all trials are provided in Supplementary Table S2, including information on trial design (including entry number, replication number, control variety number, and total number of trial plots), trial location (country, latitude and longitude), sowing date, trial infection type (and pathogen isolate information where relevant), soil type, and the crops grown on the trial site in the previous 1–3 years. Trials were grown following standard local agronomic practices, but without the application of fungicides active against the target diseases. Disease infection was scored visually at the plot level on between 1–3 timepoints in the season, depending on the trial, scored between the end of booting (growth stage 45–49; Zadoks et al. 1974) and the hard-dough stage (growth stage 87). Scores were recorded using either percentage infection, or via a 1–9 scale that was subsequently converted to percentage infection. Summary statistics (mean, median, standard deviation, and variance) were calculated using GenStat 19th edition (VSN International). Best linear unbiased estimates (BLUEs) were calculated using a linear mixed approach in REML using GenStat. For subsequent GWAS, all disease scores were transformed as log_10_(value + 1). Broad sense heritability (*H*^2^) was estimated using the method of Cullis et al. (1996).

**Table 1:** Summary of winter-sown disease field trials

Trial code	Trial operator	Country	Year	Disease score (S) number	Inf. range (%)	Inf. mean (%)	Heritability (*H*^2^)
*Yellow rust trials*							
YR_1	DSV	UK	2012	YR_1_S1	0–38.5	4.9	0.77
				YR_1_S2	0–76.0	10.8	0.87
YR_2	ELS	UK	2012	YR_2_S1	0–73.8	8.4	0.90
				YR_2_S2	0–100	13.2	0.94
YR_3	LSW	SWE	2012	YR_3_S1	0–55.7	6.9	0.51
				YR_3_S2	0–100	36.8	0.85
YR_4	LIM	UK	2012	YR_4_S1	0–79.3	7.5	0.92
				YR_4_S2	0–100	14.9	0.95
YR_5	SEJ	DNK	2012	YR_5_S1	0–77.0	5.9	0.71
				YR_5_S2	0–100	13.2	0.93
YR_6	SEJ	DNK	2013	YR_6_S1	0–92.8	10.3	0.90
				YR_6_S2	0–100	19.9	0.94
YR_7	ELS	UK	2014	YR_7_S1	0–100	8.4	0.90
YR_8	JIC M	UK	2014	YR_8_S1	0–75.0	2.6	0.83
YR_9	JIC B	UK	2014	YR_9_S1	0–76.5	4.0	0.73
YR_10	JIC T	UK	2014	YR_10_S1	0–50.0	2.3	0.20
YR_11	RAGT	UK	2014	YR_11_S1	0–50.4	2.1	0.84
YR_12	SYN	UK	2014	YR_12_S1	0–100	11.8	0.97
YR_13	SYN	FRA	2014	YR_13_S1	0–100	10.5	0.89
				YR_13_S2	0–100	16.8	0.88
*Other trials with yellow rust scores*							
BR_2	NOR	DEU	2012	BR_2_YR_S1	0–20.5	0.3	0.67
ST_4	SYN	UK	2014	ST_4_YR_S1	0–100	12.6	0.38
PM_4*	NIAB	UK	2012	PM_4_YR_S1	0–60.5	6.0	0.88
				PM_4_YR_S2	0–80.6	8.9	0.93
*Brown rust trials*							
BR_1^†^	LSW	DEU	2012	BR_1_S1	0–6	0.7	0.26
				BR_1_S2	0–32.7	4.7	0.43
				BR_1_S3	0–36.3	11.2	0.38
BR_2	NOR	DEU	2012	BR_2_S1	0–12.3	1.5	0.5
BR_3	RAGT	UK	2012	BR_3_S1	0–51	1.0	0.86
				BR_3_S2	0–50.5	1.0	0.81
BR_4	DSV	DEU	2013	BR_4_S1	0–18	5.9	0.64
				BR_4_S2	0–50.4	9.5	0.66
BR_5^†^	KWS	UK	2013	BR_5_S1	0–27.5	0.4	0.15
BR_6	LSW	DEU	2013	BR_6_S1	0–68.3	10.0	0.64
BR_7	RAGT	UK	2013	BR_7_S1	0–40.5	2.9	0.83
				BR_7_S2	0–76.8	8.4	0.86
				BR_7_S3	0–91.7	17.3	0.88
*Other trials with brown rust scores*							
PM_4*	NIAB	UK	2012	PM_4_BR_S1	0–10.2	0.8	0.80
*Septoria tritici blotch trials*							
ST_1^†^	ELS	UK	2012	ST_1_S1	2.70–99.7	46.5	0.12
ST_2^†^	KWS	UK	2012	ST_2_S1	2.1–98.7	44.7	0.27
ST_3	LIM	UK	2012	ST_3_S1	0–92.1	21.6	0.62
ST_4^†^	SYN	UK	2012	ST_4_S1	0.1–25.0	5.9	NA (1 rep)
ST_5	KWS	UK	2013	ST_5_S1	0.6–34.0	6.2	0.6
				ST_5_S2	1.9–73.9	16.4	0.4
ST_6^†^	LIM	UK	2013	ST_6_S1	0–60.3	13.2	0.61
ST_7^†^	SEJ	DNK	2013	ST_7_S1	20.5–74.7	51.8	0.65
ST_8	KWS	UK	2014	ST_8_S1	1.3–74.3	26.2	0.61
*Other trials with Septoria tritici blotch scores*							
BR_3^†^	RAGT	UK	2012	BR_3_ST_S1	0–81.8	24.5	0.63
YR_11	RAGT	UK	2014	YR_11_ST_S1	0–15.2	4.4	0.67
*Powdery mildew trials*							
PM_1	SEJ	DNK	2014	PM_1_S1	0–25.0	1.0	0.85
PM_2	JIC	UK	2015	PM_2_S1	0–39.6	6.1	0.62
				PM_2_S2	0–77.6	14.6	0.69
				PM_2_S3	0–79.7	21.3	0.70
				PM_2_S4	0–79.7	26.3	0.71
				PM_2_S5	0–89.5	32.0	0.66
*Other trials with powdery mildew scores*							
BR_4	DSV	DEU	2013	BR_4_PM_S1	0–50.3	6.6	0.81
ST_5	KWS	UK	2013	ST_5_PM_S1	0.4–75	10.9	0.82
ST_6^†^	LIM	UK	2013	ST_6_PM_S1	0–67.5	1.5	0.66

Year designation = harvest year

^*^No powdery mildew infection occurred in this trial

^†^No significant genome-wide association study (GWAS) hits identified

*Inf.* infection

*DEU* Germany, *DNK* Denmark, *FRA* France, *SWE* Sweden, *UK* United Kingdom

### Statistical analysis

Principal coordinate analysis (PCoA) was conducted in R using the package ape (Paradis and Schliep 2019) with 3563 markers that had been ‘skimmed’ to remove a SNP in each pair with an absolute correlation of *r* ≥ 0.7. Linkage disequilibrium was estimated as the *r*^2^ between all pairs of unique anchored SNPs (8962) using the R package sommer (Covarrubias-Pazaran 2016). The LD decay was determined by plotting the *r*^2^ values against physical distance (Mbp), and for each of the A, B and D subgenomes a trend line was calculated by locally weighted polynomial regression (LOESS) curve in R. The physical distance of LD decay to a threshold of *r*^2^ = 0.2 was inspected for each genome. GWAS was performed using the R package GWASpoly (Rosyara et al. 2016), which identified marker-trait associations using the Mixed Linear Model (MLM) (Yu et al. 2006). The GWAS accounted for population structure (principal components = 5) and kinship as fixed and random effects, respectively. Using GWASpoly, the kinship matrix was calculated using a subset of 4023 SNPs ‘skimmed’ from the 11,858 mapped and unmapped SNPs to remove a marker in each pair with an absolute *r* ≥ 0.75. The significance of marker–trait associations was determined using two thresholds: (1) the false discovery rate (FDR) (Benjamini and Hochberg 1995) using a *q* value cut-off of *q* = 0.05 and (2) the permutation threshold (Churchill and Doerge 1994), using 1000 permutations and *α* = 0.05. In cases where the FDR threshold was too lenient (under 2.9) just the permutation threshold was used. Markers in Manhattan plots were ordered according to the anchored physical positions from the wheat reference genome, with unmapped markers at the end. Covariate variables were included in successive iterations of GWAS. Marker–trait associations (MTAs) were consolidated into discrete quantitative trait loci (QTL) by taking the mapped significant markers, organising them by physical and genetic distance, and choosing QTL cut-offs by taking into account linkage disequilibrium decay. QTL were named using the highest scoring physically mapped marker in the defined region. GWAS results were subsequently drawn in a chromosomal ideogram using R package LinkageMapView (Ouellette et al. 2018). Replicated GWAS hits between two or more diseases that were located within 25 Mbp of each other were termed here ‘multi-resistance loci’ (this interval was arbitrarily set). Power analyses were undertaken using previously described methods (Wright et al. 2021), using simulated phenotypes with different *H*^2^ (0.25, 0.50, 0.75, 0.90 or 0.99) and simulated focal QTL explaining different amounts of the variance (5%, 10%, 25%, 50% and 100%). For each combination of *H*^2^ and percentage variance, 1000 simulations were run.

### Validation of GWAS hits

A subset of the SNPs identified as significant in our GWAS analysis were converted from the 90 k array to the Kompetitive Allele-Specific PCR (KASP) platform (LGC Genomics, UK) for subsequent use for validation via independent bi-parental populations, termed BP1 to BP8, provided by the breeding companies involved. KASP primer design was undertaken using PolyMarker (Ramirez-Gonzalez et al. 2015), with primers listed in Supplementary Table S3. DNA for KASP genotyping was extracted from a set of 95 cultivars selected from the GWAS panel using the DNEasy Kit (Qiagen) and KASP genotyping undertaken using KASP V4.0 2 × Master Mix (LGC Biosciences) using a ProFlex PCR System Thermocycler (Applied Biosystems) with the following settings: 1 cycle at 94 °C for 15 min; 10 cycles at 94 °C for 20 s, 65 °C for 60 s with a touchdown of − 0.8 °C/cycle to 57 °C; 35 cycles at 94 °C for 20 s, 57 °C for 60 s; final hold at 10 °C. Fluorescence of VIC and FAM fluorophore 5’ end labelled PCR products were subsequently read using a Scientific QuantStudio™ 12 K Flex Real-time PCR System (Thermo Fisher Scientific). ROX was used as a passive fluorescent reference to allow normalisation of variations in signal caused by differences in well-to-well liquid volume, following the manufacturer’s instructions (LGC Genomics). Results were visualised using SNP Viewer v.1.99 (http://lgcgenomics.com/). KASP markers confirmed as co-dominant were used to validate GWAS hits in bi-parental populations constructed either by single seed descent, or by the doubled haploid approach. Boxplots showing the distribution of the resistant and susceptible alleles and percentage of yellow rust infection recorded from field trials undertaken in the UK (using populations BP1, BP3, BP5-BP7), France (BP1), Denmark (BP2, BP4) and Germany (BP8) in 2015 were plotted using ggplot2 (Wickham 2016) and significance tested via a one-way ANOVA in R. To analyse significance per QTL across trials, a two-way ANOVA was performed with independent variables of KASP score and experiment. For YR_2A010, the two KASP markers used were treated as the same as there was no evidence in our datasets that the introgression has been broken up by recombination; for YR_6A610, when KASP was added into the model, the effect was not significant. Trials where there were on average less than 5% yellow rust infection were excluded from the validation set.

### Haploblock and pedigree analysis

Genotypic data were used to create haploblocks and their corresponding haplotypes using Haploview v4.2 (Barrett et al. 2005) with additional manual curation. Where required, genotype calls at SNPs defining the haploblock were also determined in the genome assembly of *T. aestivum* cultivar ‘Jagger’ (Walkowiak et al. 2020) via BLASTn using Ensembl Plants (Yates et al. 2022). Plots of the wheat pedigree were constructed with Helium v1.19.09.03 (Shaw et al. 2014) using the pedigree published by Fradgley et al. (2019).

## Results

### Characteristics of the association mapping panel: population substructure, linkage disequilibrium and experimental power

We assembled a wheat association mapping panel, termed here the ‘WAGTAIL’ panel. It consisted of 480 European wheat cultivars released across 10 countries between 1916 and 2007. The cultivars predominantly originated from the United Kingdom (UK, 70%), France (12%) and Denmark (8%), and the majority were winter type (93%) (Supplementary Table S1). Genotyping the panel with a 90,000 feature SNP array resulted in 26,015 polymorphic genetic markers (see Supplementary Text 1 for additional details). After removing 359 markers with > 10% missing data and 124 markers with a minor allele frequency > 2.5%, 25,532 markers remained. Of these, we were able to anchor 20,166 markers to the wheat physical map, leaving 5366 unmapped markers. Duplicated SNPs (based on 100% correlation) were then removed from both the mapped and unmapped datasets, resulting in 8962 mapped markers and 2896 unmapped markers. Therefore, the final SNP data-matrix consisted of 11,858 markers genotyped across 480 cultivars (Supplementary Table S1).

**Fig. 1:**
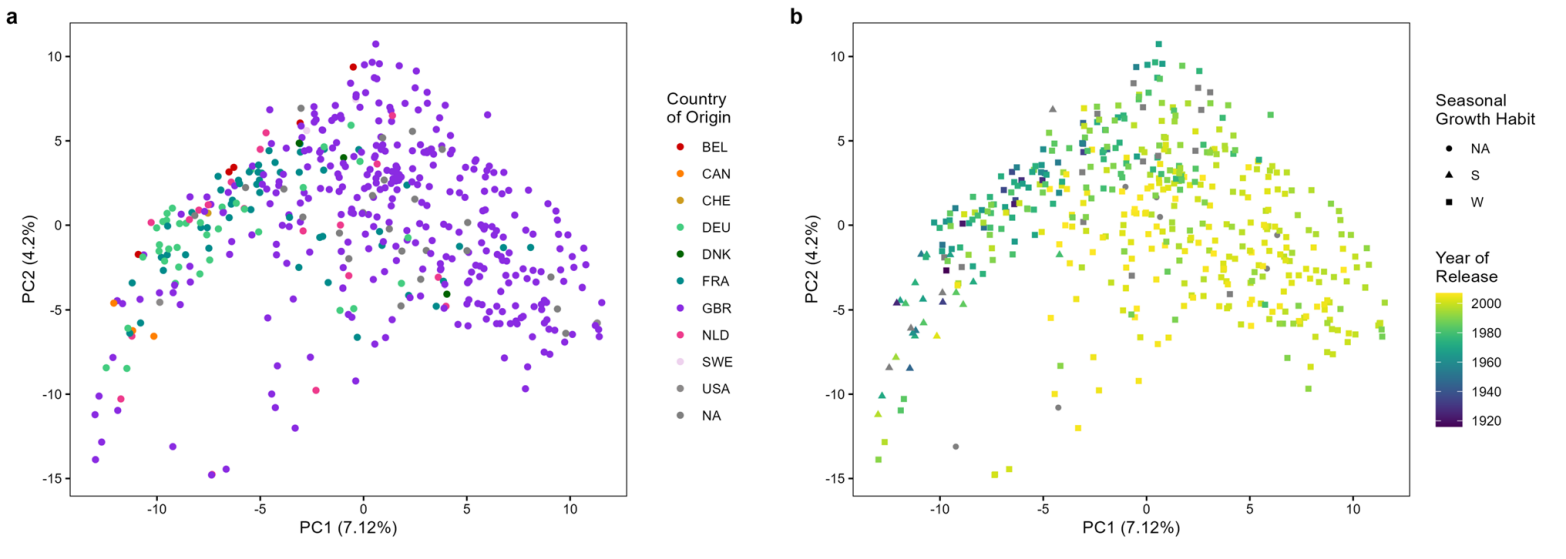
Principal coordinate analysis of the WAGTAIL association mapping panel. A subset of 3563 markers were used, ‘skimmed’ from the overall marker set to remove a SNP in every pair with an absolute *r*^2^ ≥ 0.7. The two principal coordinates are shown, overlaid with **a** country of origin, and **b** the year of release and seasonal growth habit (spring, S ▲; winter, W ■)

Mean genetic marker density for the A and B subgenomes was similar, at 1.55 and 2.05 markers/Mbp, but was lower on the D subgenome (0.64 markers/Mbp). Genetic marker number per chromosome ranged from 2196 (chromosome 1B) to 85 (chromosome 4D) (Supplementary Table S4). Principal coordinate analysis (PCoA) identified relatively limited genetic substructure (Fig. 1), with the first two principal coordinates (PCs) accounting for 11.3% of the variation (PC1 = 7.1%, PC2 = 4.2%). While the majority of the cultivars in the panel were from the UK, the German (DEU) and Dutch (NLD) cultivars formed clusters in the PCoA plots. Overlaying ‘winter’ and ‘spring’ seasonal growth habit designations found spring cultivars to form a loose subcluster within the overall plot, determined predominantly by PC1. Furthermore, year of cultivar release showed a notable visual trend for newer varieties to be further separated from the spring cultivars in PCoA space.

**Fig. 2:**
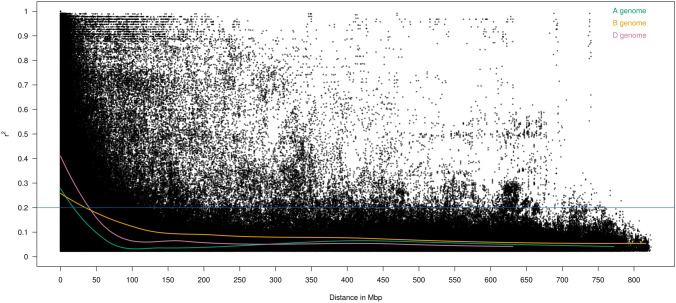
Linkage disequilibrium (LD) decay plot for the wheat association mapping panel (*n* = 480). Pairwise correlation (*r*^2^) between markers (8962) on all 21 wheat nuclear chromosomes was calculated as a metric for LD. The locally estimated scatterplot smoothing (LOESS) curves summarising LD on the A (green), B (yellow) and D (pink) subgenomes are shown. The blue horizontal line indicates the *r*^2^ threshold of 0.2

**Fig. 3:**
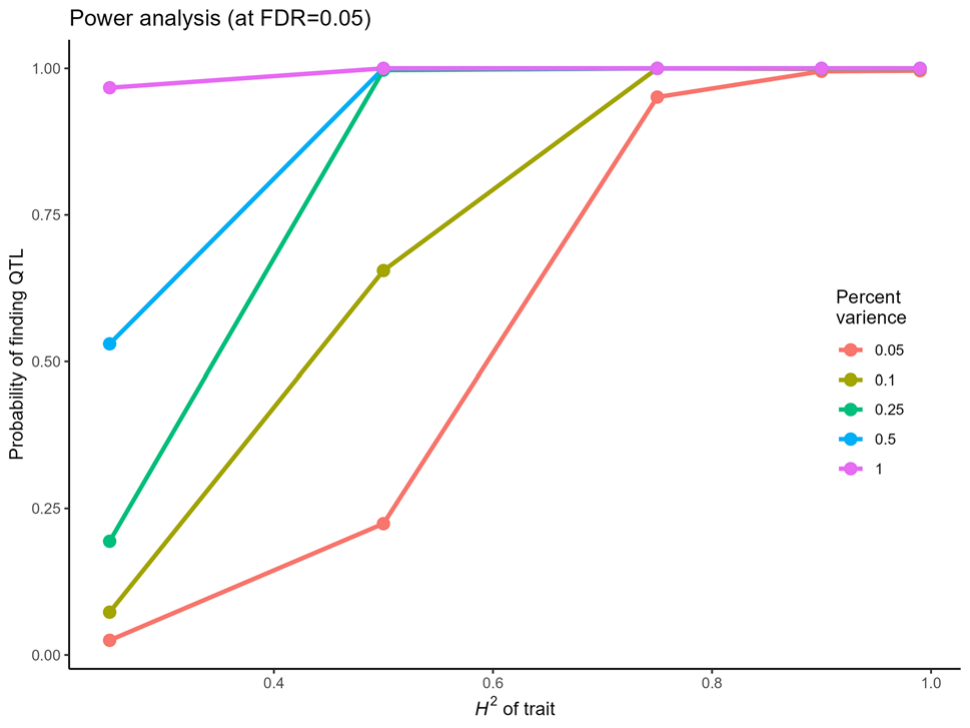
Power analyses conducted with the WAGTAIL association mapping panel. Five heritability values were used to simulate phenotypes linked to quantitative trait loci (QTL) explaining four different amounts of phenotypic variance. For each combination of heritability and explained phenotypic variance, 1000 simulations were completed and the frequency of how many times the focal QTL was found above the FDR threshold of *q* = 0.05 in each association mapping scan determined the probability of finding QTL

Using the 8962 skimmed and anchored marker set, we then investigated the distribution and extent of linkage disequilibrium within all chromosomes via linkage disequilibrium decay plots, with a trend line for each subgenome A, B and D calculated by locally weighted polynomial regression (LOESS) (Fig. 2). The intersection between the LOESS curve and *r*^2^ = 0.2 suggested that linkage disequilibrium decayed at a relatively low rate within chromosomes, returning distances for the A, B and D subgenomes of 20, 36 and 41 Mbp, respectively. To further explore the suitability of the panel for genome-wide association studies, we used our data-matrix of 480 cultivars and 8962 SNPs to undertake power analyses, whereby the probability of identifying a simulated QTL was investigated when heritability (*H*^2^) and percentage variance explained by the QTL were varied (Fig. 3). As the percentage variance explained by the QTL and *H*^2^ increased, the probability of finding the QTL increased. Where *H*^2^ was high (≥ 0.75), the probability of QTL detection was close to 1.0 irrespective of the percent variance explained. At more modest levels of *H*^2^ (0.50) and when the percentage variance explained by the QTL was ≥ 10%, the probability of QTL detection remained relatively high (> 66%). However, the probability of identifying QTL fell to below 20% when low values for both heritability (*H*^2^ = 0.25) and percent variance explained (≤ 0.25) were modelled. For QTL explaining 5% of variance, a 50% detection ability was achieved when *H*^2^ was over about 0.6.

### Disease resistance phenotyping

**Fig. 4:**
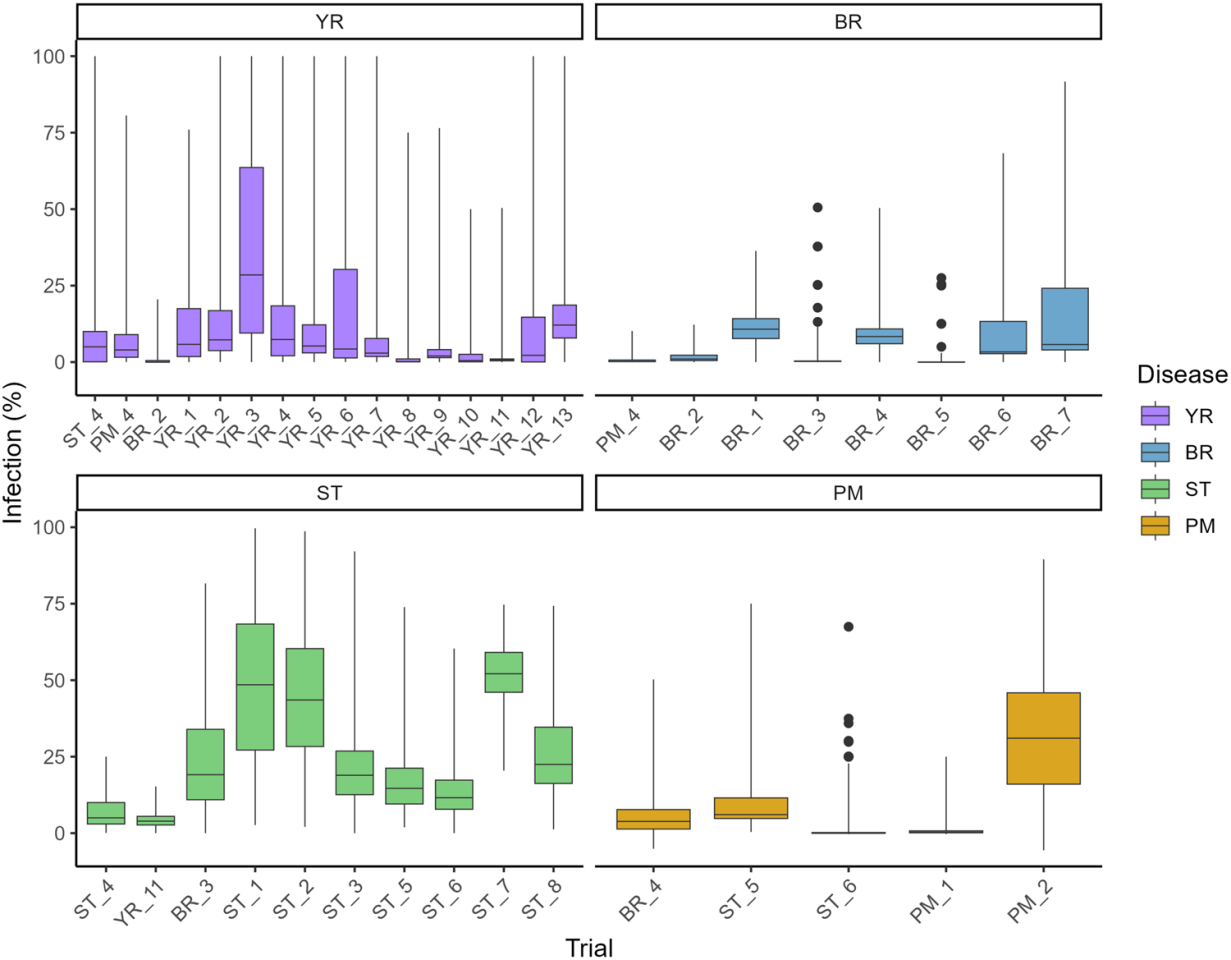
Boxplots showing percentage disease infection in the WAGTAIL association mapping panel as assessed on the last scoring date measured at each field trial. Values represent untransformed Best Linear Unbiased Estimates (BLUEs). Trials are arranged by year (between 2012 and 2015), with trial abbreviations as listed in Table 1. The line within the box represents the median, bottom and top of boxes represent upper and lower quartiles and lines below and above box minimum and maximum values, respectively. Dots show outliers. YR = yellow rust. BR = brown rust. ST = Septoria tritici blotch, PM = powdery mildew

We used the association mapping panel to conduct a total of 31 disease assessment trials between 2012 and 2015, located in the UK, Germany, Denmark, Sweden and France. Of these trials, 13 were for yellow rust (YR), seven for brown rust (BR), eight for Septoria tritici blotch (ST) and three for powdery mildew (PM) (Table 1). In some trials, more than one disease was scored where the opportunity arose and where both diseases could be scored without mutual interference, and in one PM trial no PM infection occurred, such that in total 39 disease–trial combinations and 58 disease–trial–timepoint combinations were scored (Table 1). For yellow rust and brown rust, where percentage disease infection scores were measured in all trials on two or three separate dates (indicated in the trait names using the postscript ‘_S1’, ‘_S2’ or ‘_S3’), the percentage infection at later score dates were consistently higher than on the first. Overall, Septoria tritici blotch showed the highest infection based on mean percentage infection across all trials (25.9%), followed by yellow rust (11.2%), powdery mildew (10.6%) and brown rust (6.5%). Boxplots for disease resistance at the last score date for each trial–trait–score date combination are shown in Fig. 4. For the majority of YR, BR, ST and PM datasets, percentage disease was skewed towards low infection—accordingly, best linear unbiased estimates (BLUEs) were log-transformed for all downstream analyses (Supplementary Table S5). Heritabilities for yellow rust trials were mostly high (20/24 disease–trial–timepoint combinations *H*^2^ > 0.7). For brown rust, heritability was variable (6/14 disease–trial–timepoint combinations *H*^2^ > 0.7, 5/14 combinations *H*^2^ ≤ 0.5). For Septoria, heritability for 0/11 disease–trial–timepoint combinations were *H*^2^ > 0.7 and 3/11 were *H*^2^ < 0.5) (Table 1). For powdery mildew, 5/9 disease–trial–timepoint combinations were *H*^2^ ≥ 0.7. For each disease, there was significant positive correlation between variety means from trial–trait–score combinations for the majority of comparisons investigated (Supplementary Fig. S1). Correlations within disease datasets were highest for yellow rust datasets and powdery mildew datasets, for which all comparisons were significant at *P* ≤ 0.001. Disease score correlations between all but one Septoria tritici blotch trial–trait–score combinations were significant at *P* ≥ 0.05. For brown rust, while the majority of correlations were significant at *P* ≤ 0.001, non-significant positive correlations were identified for four trial comparisons (BR_1/BR_7, BR_1/BR_3, BR_2/BR_3 and BR_2/BR_5). Several notable inter-disease correlations were observed. This included positive significant correlations (*P* ≤ 0.05) between yellow rust and powdery mildew (53 of 80 comparisons, 66%), yellow rust and brown rust (20 of 128 comparisons, 16%) and brown rust and powdery mildew (18 of 40 comparisons, 45%). Conversely, significant negative correlations between trial–trait–score date combinations were notable between subsets of Septoria trials and those for powdery mildew (14 of 50 comparisons, 28%) and brown rust (20 of 80 comparisons, 25%). Notably, most of the significant negative correlation (*P* ≤ 0.05) reported between Septoria and brown rust trials originated via comparisons with three brown rust trials (BR_3, BR_4, BR_6, 18 of 30 trial comparisons; Supplementary Fig. S1).

### Marker–trait associations

**Fig. 5:**
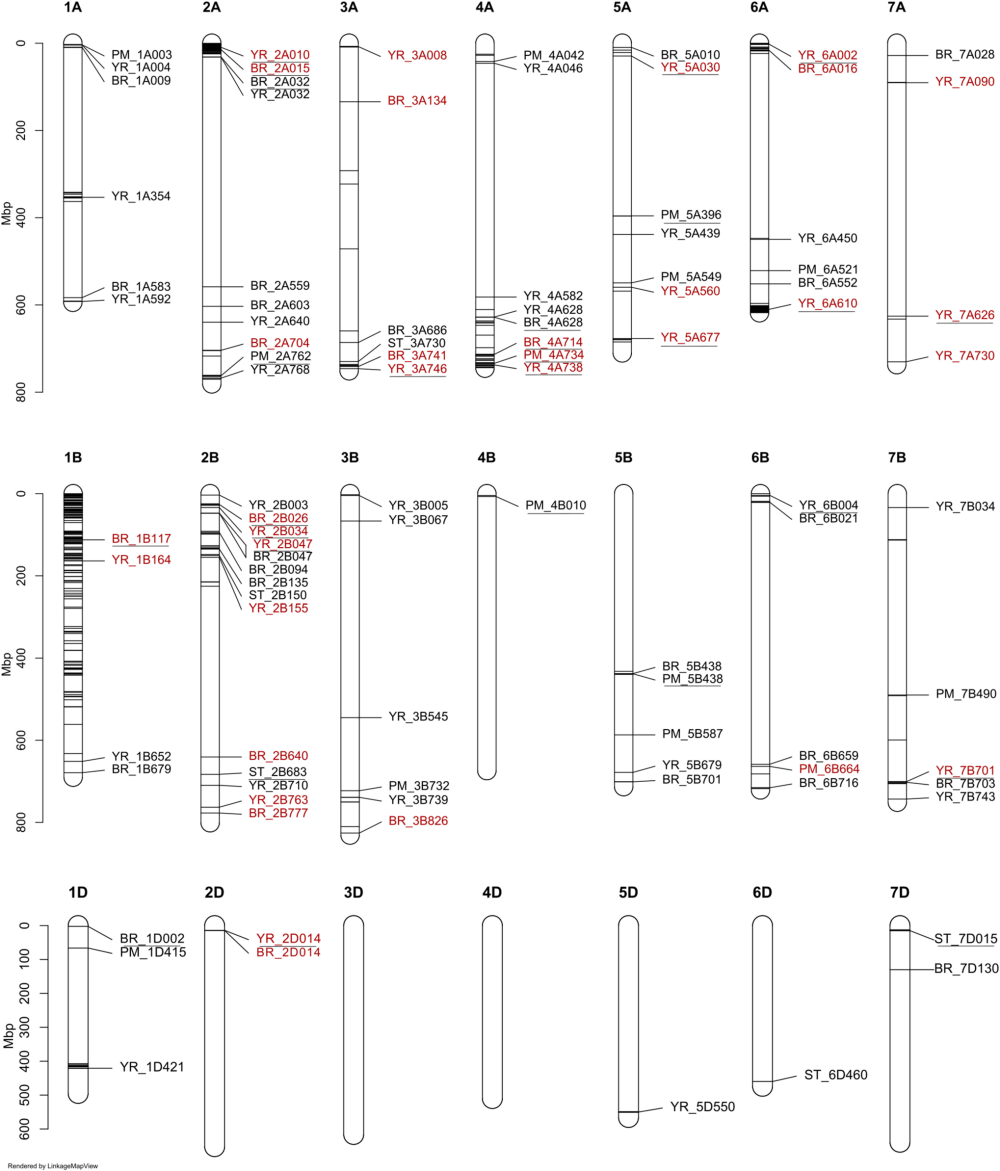
Chromosomal ideogram showing the distribution across the wheat reference genome of all genetic resistance loci identified via genome-wide association studies (GWAS). Black horizontal lines within each chromosome represent positions of those genetic markers significant above the false discovery rate (FDR) *q* = 0.05 significance threshold, with the physical location displayed as a scale on the left-hand side in megabase pairs (Mbp). Genetic loci identified using genetic markers significant above the FDR *q* = 0.05 threshold are labelled on the ideogram. YR: yellow rust, BR: brown rust, PM: powdery mildew, ST: Septoria tritici. Genetic loci identified in two or more trials are highlighted in red, and those with one or more instances of significance above the permutated *α* = 0.05 significance threshold are underlined

**Fig. 6:**
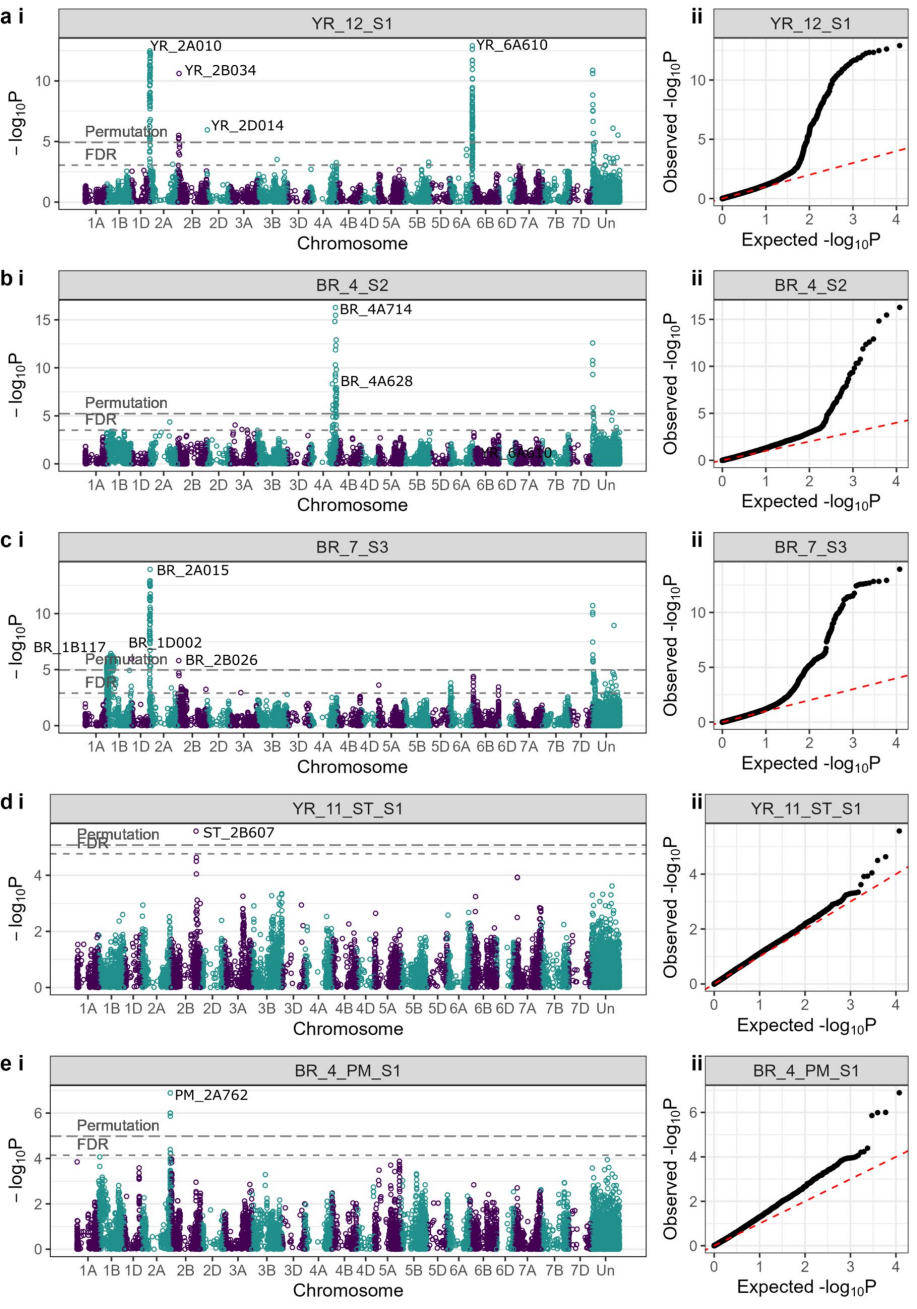
Manhattan plots (i) and quantile–quantile (Q–Q) plots (ii) showing examples of the results of genome-wide association study (GWAS) analysis of the four disease resistance traits scored in our wheat association mapping panel. For Manhattan plots, each datapoint represents a single marker–trait association (MTA); markers are plotted using physical map location on the wheat reference genome. Two significance thresholds are shown: the false discovery rate (FDR) *q* = 0.05 threshold, and the more stringent *α* = 0.05 threshold as determined via permutation. Genetic loci above the permutation threshold are labelled on each Manhattan plot. **A** Disease score 1 from yellow rust resistance trial YR_12, undertaken in the UK in 2014. **B** Disease score 2 from brown rust resistance trial BR_4, undertaken in Germany in 2013. **C** Disease score 3 from brown rust resistance trial BR_7, undertaken in the UK in 2013. **D** Septoria tritici blotch disease score 1 from trial YR_11, undertaken in the UK in 2014. **E** Disease score 1 for powdery mildew resistance, as scored in brown rust trial BR_4, undertaken in Germany in 2013. Q–Q plots compare observed (O) versus expected (E) significance values. The red dashed line denotes where O=E. All results shown are based on log-transformed phenotypic data

We used the 58 trial–disease–timepoint combinations (subsequently termed here, ‘traits’) to undertake genome-wide association studies (GWAS), initially employing a false discovery rate significance threshold of FDR = 0.05. GWAS identified a total of 2054 marker–trait associations (MTAs), of which 1702 involved genetically mapped markers and 352 involved unmapped markers (Supplementary Table S6). Considering the mapped markers only, and taking into account LD decay, physical and genetic distance, these MTAs resolved into the following number of distinct genetic loci: 43 for YR, 34 for BR, 5 for ST, and 14 for PM (Fig. 5) (Supplementary Table S7, Supplementary Fig. S2). For each disease, GWAS hits with the highest significance are shown in Fig. 6a–e (Manhattan plots for all GWAS analyses are shown in Supplementary Fig. S2).

Yellow rust: GWAS hits were identified in all 16 field trials in which yellow rust was scored (Fig. 5) (Supplementary Table S7). The majority of loci located on the A and B subgenomes (23 and 17, respectively), while just three were found on the D subgenome. The highest number of YR resistance loci was located on chromosomes 2B (6), 2A (4), 3B (4), 4A (4) and 5A (4). Of the 43 yellow rust resistance genetic loci which were statistically significant in at least one disease trial, 20 were identified in two or more trials (Table 2; highlighted in red in Fig. 5) of which 12 were identified using the more stringent permutated *P* = 0.05 significance threshold (underlined in Fig. 5). The most replicated YR resistance locus was on chromosome 4A at ~ 738 Mbp (termed here, YR_4A738), which was identified in eight separate trials between 2012 and 2014 (five in the UK, two in Denmark and one in France). Overall, the YR resistance loci with the highest significance values (− log_10_* P* ≥ 8.21) were identified in the UK in 2014: YR_2A010 on the short arm of chromosome 2A (identified in five trials) and YR_6A610 on the long arm of chromosome 6A (identified in six trials) (Fig. 6a). These two loci had very high significance values (Fig. 6a) but relatively small SNP effect sizes (Table 2).

**Table 2:** Summary of the 39 significant disease resistance genetic loci identified via genome-wide association study (GWAS) in two or more trials

Genetic locus	Multi-resistance locus	Chr	Interval (Mbp)	Peak SNP name	Peak SNP pos. (Mbp)	Peak SNP sig. (− log_10_P)	Effect on pheno (% inf.)	No. of trials
*Brown rust (total no. trials* = *8)*								
BR_1B117		1B	1.20–11.31	*BS00009715_51*	112.4	6.44	− 0.50	5
BR_2A015	MT25Mb_1	2A	2.23–24.28	*BS00004089_51*	14.8	13.95	− 0.67	2
BR_2A704		2A	703.98–717.17	*Excalibur_c40617_983*	704.0	3.58	0.83	2
BR_2B026	MT25Mb_2	2B	26.30–33.84	*Kukri_c40764_367*	26.3	5.80	− 0.48	2
BR_2B640		2B	214.59–640.79	*BS00069685_51*	640.8	3.83	− 0.36	2
BR_2B777	MT25Mb_3	2B	777.33	*RAC875_c19685_944*	777.3	3.57	0.47	2
BR_2D014	MT25Mb_4	2D	14.40	*BobWhite_c15073_502*	14.4	3.34	− 0.27	2
BR_3A134		3A	134.25–471.36	*BobWhite_c35303_192*	134.2	4.04	− 0.44	2
BR_3A741	MT25Mb_5	3A	736.39–741.24	*Tdurum_contig5009_349b*	741.2	3.61	− 0.28	2
BR_3B826		3B	810.33–826.1	*Tdurum_contig42131_1300*	826.1	3.47	1.18	2
BR_4A714	MT25Mb_6	4A	698.04–743.98	*BobWhite_c20306_111*	713.5	16.27	− 0.75	2
BR_6A016		6A	11.41–23.72	*Excalibur_rep_c105463_330*	15.7	4.30	− 0.38	3
*Powdery mildew (total no. trials* = *5)*								
PM_4A734	MT25Mb-6	4A	610.49–742.09	*BS00110758_51a*	734.0	5.22	− 0.62	2
PM_6B664		6B	663.68–681.92	*RAC875_c5129_280*	663.7	5.04	0.68	2
*Yellow rust (total no. trials* = *16)*								
YR_1B164		1B	14.85–163.78	*Excalibur_rep_c92475_275*	163.8	4.82	0.26	2
YR_2A010	MT25Mb_1	2A	0.40–24.28	*wsnp_Ra_c8771_14786376b*	9.5	12.48	− 0.77	5
YR_2B034	MT25Mb_2	2B	24.91–33.84	*BobWhite_rep_c64429_660a*	33.8	10.61	1.96	4
YR_2B047		2B	47.43–47.43	*BS00041587_51*	47.4	5.95	0.81	6
YR_2B155		2B	133.7–154.99	*Kukri_c36783_91*	155.0	4.17	− 0.57	2
YR_2B763	MT25Mb_3	2B	0.00–763.09	*BS00070301_51a*	763.1	4.54	− 0.30	2
YR_2D014	MT25Mb_4	2D	0.00–13.99	*RAC875_c90426_151*	14.0	5.96	1.24	3
YR_3A008		3A	7.43–8.87	*BS00037189_51*	7.8	4.86	0.73	2
YR_3A746	MT25Mb_5	3A	746.17	*wsnp_Ex_c60462_60905848*	7746.2	5.69	− 0.68	2
YR_3B739		3B	738.75–750.36	wsnp_Ex_rep_c101457^*^	739.1	4.85	− 0.48	2
YR_4A738	MT25Mb_6	4A	734.00–738.78	*Excalibur_c65272_341*	737.5	6.70	− 0.62	8
YR_5A030		5A	15.85–29.51	*Excalibur_rep_c90275_262*	29.5	6.07	0.77	2
YR_5A560		5A	559.52–568.27	*BS00021955_51*	559.5	4.21	− 0.60	2
YR_5A677		5A	677.13–684.94	*BS00022867_51*	677.1	5.07	− 0.46	4
YR_6A002		6A	0.29–13.07	*Excalibur_c50323_215*	1.9	5.83	− 0.44	5
YR_6A610		6A	596.58–11.13	*GENE-4021_496*	610.3	12.91	0.54	6
YR_7A090		7A	89.84–90.65	*Kukri_c5757_530*	90.7	4.17	1.23	2
YR_7A626		7A	625.74–632.59	*BS00105558_51*	625.7	5.73	− 0.48	3
YR_7A730		7A	730.43–730.43	*Kukri_c11451_1882*	730.4	4.97	− 0.63	2
YR_7B701		7B	700.57–705.73	*Excalibur_c7338_563*	700.8	6.04	0.51	3

The six multi-resistance genetic loci in which replicated resistance loci were identified for two or more diseases within 25 Mbp are listed as MT25Mb_1 to _6. Chromosome locations are based on the wheat reference genome (IWGSC 2018). Effect on phenotype is indicated as change in disease leaf infection percentage conferred by the reference allele

^*^Complete marker name: *wsnp_Ex_rep_c101457_86817938*

*Chr.* chromosome, *No*. number, *Pheno.* phenotype, *Pos.* position

Brown rust: Of the eight trials undertaken in whcih BR was scored (four in the UK and four in Germany), GWAS hits for BR resistance were identified in six trials (Fig. 5) (Supplementary Table S7). No significant associations were identified in trials BR_1 (Germany 2012, low BR infection pressure) and BR_5 (UK, 2012) (Table 1). The 34 BR resistance genetic loci identified were distributed predominantly on the A and B subgenomes (16 and 15 loci, respectively), with just three found on the D subgenome. The group 2 chromosomes possessed notably high numbers: five for chromosome 2A and six for chromosome 2B. Twelve of the 34 BR resistance loci were replicated in two or more trials (Table 2; Supplementary Table S7; highlighted in red in Fig. 5), with two loci being by far the most significant (− log_10_ *P* value above the FDR = 0.05 significance threshold > 11): BR_2A015 on chromosome 2A at ~ 15 Mbp) and BR_4A714 on chromosome 4A at ~ 714 Mbp (Fig. 6b). The BR resistance genetic locus identified in the highest number of trials was BR_1B177 on chromosome 1B at ~ 177 Mbp, found in five trials in Germany and the UK in 2012 and 2013 (trials BR_3, BR_4, BR_6, BR_7, PM_4).

Septoria tritici blotch: GWAS hits for Septoria tritici blotch (ST) resistance were identified in three of the ten trials scored: ST_3 (UK, 2012), ST_5 (UK, 2013) and YR_11_ST (UK 2014) (Fig. 5) (Supplementary Table S7). Five genetic loci were identified, distributed across the A (1 loci), B (2) and D (2) subgenomes. Of these loci, none were replicated (Table 2; Fig. 5). The most significant ST resistance locus was ST_7D015, located on the short arm of chromosome 7D at ~ 15 Mbp, identified in trial ST_5 (UK, 2013).

Powdery mildew: GWAS hits were identified in all five field trials in which powdery mildew infection was scored (Fig. 5) (Supplementary Table S7). In total, 14 genetic loci were identified across 11 chromosomes. Resistance loci were more common on the A and B subgenomes (7 and 6 loci, respectively) than on the D subgenome (1 locus), with chromosome 4A, 5A and 5B possessing two resistance QTL each. Two replicated resistance loci were identified, PM_4A734 in trials PM_1 and PM2, and PM_6B664 in trials PM_2 and ST_6 (Table 2; highlighted in red in Fig. 5). The most significant PM resistance loci identified were PM_2A762 (chromosome 2A at ~ 762 Mbp, identified in trial BR_4) (example Manhattan plot shown in Fig. 6e) and PM_4B010 (chromosome 4B at ~ 10 Mbp, identified in trial ST_5), both of which returned − log_10_*P* values ≥ 1.98 above the FDR = 0.05 significance thresholds applied in their relevant trials. As with ST but in contrast to YR and BR, − log_10_*P* values for even the most significant PM loci were not especially high.

### Chromosomal distribution of disease resistance genetic loci

The occurrence of disease resistance genetic loci across the genome was enriched towards chromosome ends. The few loci that were located in pericentromeric regions, as defined by comparison of the physical and genetic maps (Supplementary Table S8), were typified by large physical interval sizes, due to reduced genetic recombination (Supplementary Table S7).

**Fig. 7:**
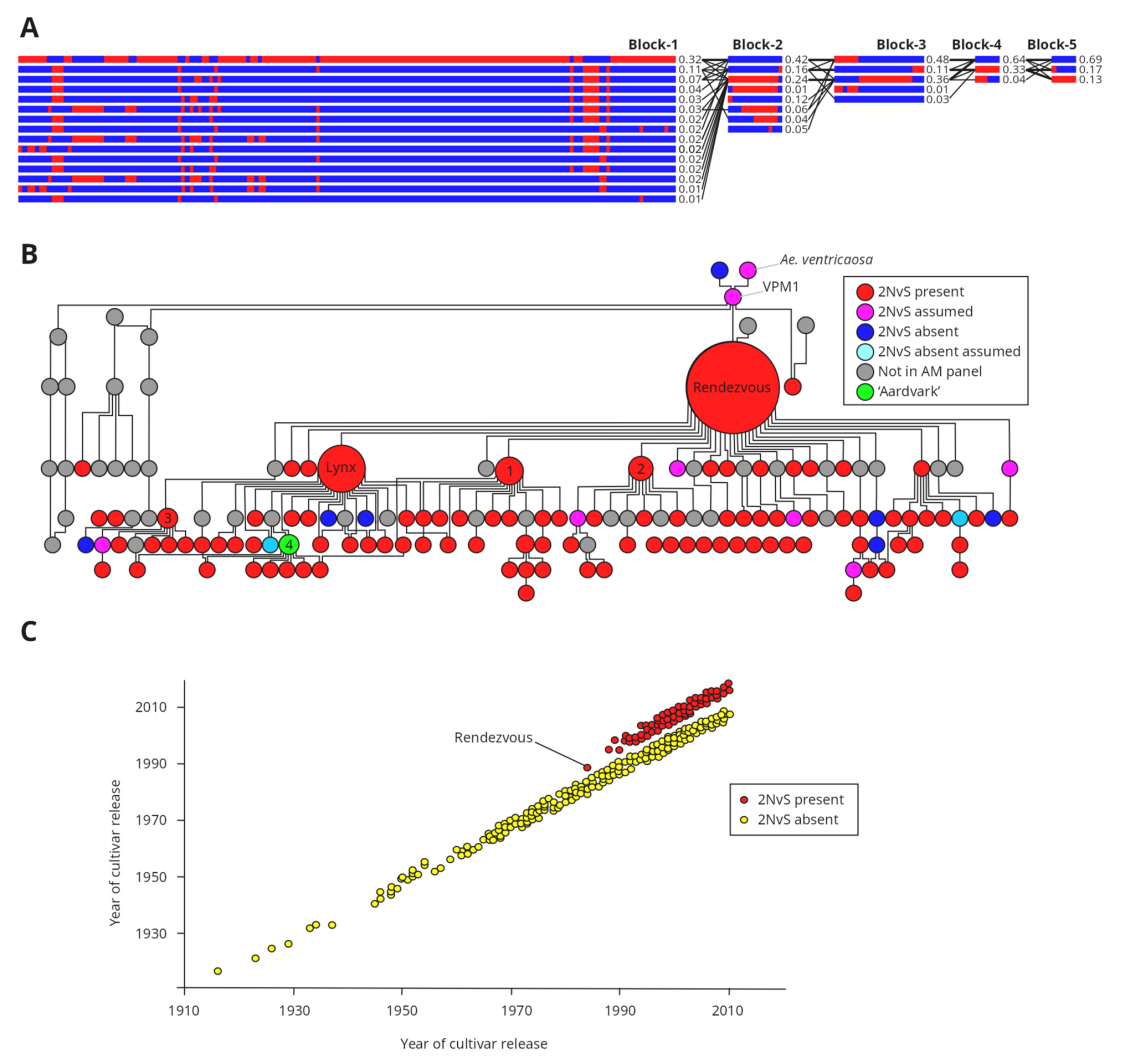
Features of the *Aegilops ventricosa* 2N^v^S chromosomal introgression in our wheat association mapping panel (*n* = 480). **A** A notably large haploblock consisting of 162 SNPs (Block 1) was identified on the short arm of chromosome 2A. The most common haplotype within this haploblock was identified in 153 of the 480 cultivars investigated (32%), including ‘Rendezvous’, the earliest 2N^v^S carrier in our panel. ‘VPM1’ is the first published 2N^v^S carrier, and ‘Rendezvous’ has ‘VPM1’ in its pedigree (‘Virtue’ x [‘Maris-Hobbit sib’ x ‘VPM1’]). **B** Inheritance of the 2N^v^S haplotype for 97 cultivars in the wheat pedigree based on the 162-SNP haplotype. For some cultivars, assumptions have been made on whether they either carry (purple) or lack (light blue) 2N^v^S based on the haplotypes of their parents (or in the case of ‘VPM1’ and its *Ae. ventricosa* parent, based on the literature). Cultivars in the pedigree not present in our association mapping panel are shown in grey. Cultivar size is proportional to the frequency of its contribution in the pedigree. 1 = ‘Hussar’, 2 = ‘Torfrida’, 3 = ‘Equinox’, 4 = ‘Aardvark’ (highlighted in green, as our data suggest the ‘Aardvark’ genotype is anomalous, agreeing with similar conclusions by Corsi et al. (2020). **C** Rocket plot, illustrating changes in occurrence of the 2N^v^S introgression over time in our GWAS panel based on genotype call at the 2N^v^S diagnostic SNP, *BobWhite_c18101_540*. Each data point represents a cultivar, plotted against year of cultivar release (plotted positions of 2N^v^S carriers are offset on y-axis for visual clarity)

Close physical linkage between the peak GWAS hits for resistance to two or more target diseases was observed, most commonly for yellow rust and brown rust (caused by related biotrophic fungal pathogens), but also with powdery mildew or Septoria tritici blotch. For example, considering replicated GWAS hits only, seven genetic loci clusters were predicted to be located within 25 Mbp of each other (termed here ‘multi-resistance loci’), of which five were within 11 Mbp (on chromosome 2A: YR_2A010/BR_2A015; chromosome 2B: BR_2B026/YR_2B034, chromosome 2D: YR_2D014/BR_2D014; Chromosome 3A: BR_3A741/YR_3A746; chromosome 6A: YR_6A002/BR_6A016) (Fig. 5; Supplementary Table S9). Notably, this included our second most significant hits for yellow rust resistance (YR_2A010) and brown rust resistance (BR_2A015), for which the most significant markers were located within ~ 5 Mb of each other on the short arm of chromosome 2A. YR_2A010 and BR_2A015 are located in a region previously reported to carry a ~ 32-Mbp introgression from *Aegilops ventricosa* chromosome 2N^v^S (Gao et al. 2021). Analysis of our GWAS panel identified five haploblocks towards the end of the short arm of chromosome 2A, encompassing 211 SNPs across ~ 37 Mbp. Of these, an unusually large haploblock consisting of 162 SNPs was present at the start of the chromosome arm (haploblock-1), within which a single haplotype was present at a frequency of 32% (153 of the 480 cultivars) (Fig. 7a) (Supplementary Table S10). Anchoring these SNPs to the genome assembly of the German wheat cultivar ‘Jagger’, previously reported as carrying the 2N^v^S introgression (Gao et al. 2021), found that all SNPs were located within the 32.53 Mbp introgressed chromosomal segment and carry the ‘Jagger’ SNP variant (Supplementary Table S10). Of these 162 SNPs, 67 were found to each uniquely serve as a tag for the extended putative 2N^v^S haplotype (Supplementary Table S10) and resulted in highly significant GWAS *P*-values for yellow rust and brown rust (≥ 9.28 above the FDR). The 2N^v^S introgression was introduced into the wheat pedigree via the cultivar ‘VPM1’ (Dyck and Lukow 1988). Cross-referencing the presence of the 2N^v^S haplotype in our association mapping panel with a recently published pedigree of European wheat (Fradgley et al. 2019) found 97 of the 153 2N^v^S haplotype carriers to have ‘VPM1’ in their known pedigree (Fig. 7b), with most of the remaining 56 cultivars lacking sufficient pedigree information detail to determine whether ‘VPM1’ was in their pedigree. Plotting the occurrence of the 2N^v^S introgression against cultivar commercial release date shows its frequency has significantly increased over time since its introduction via ‘VPM1’ in the early 1980s (Fig. 7c), with 48% of the most recent cultivars in our panel carrying the introgression (years 2008–2010).

Of the two replicated powdery mildew resistance loci, PM_4A734 was located within 20 Mbp of replicated resistance loci for yellow rust (YR_4A738) and brown rust (BR_4A714) on the long arm of chromosome 4A. No replicated resistance loci were identified for Septoria tritici blotch.

### Validation of yellow rust GWAS hits

We selected the two most significant yellow rust resistance genetic loci identified by GWAS, YR_2A010 and YR_6A610, for independent validation in a series of eight bi-parental populations (termed BP1 to BP8). Parental lines were selected so that each bi-parental population was predicted to segregate for contrasting alleles at one or both of the target resistance loci (Supplementary Table S11), based on the parental genotypes in our GWAS dataset. The populations were phenotyped for percentage yellow rust infection in the field and the target loci genotyped using KASP markers for selected 90 k array SNPs identified by GWAS in the association mapping panel (YR_2A010: SNPs *Kukri_c18149_581* and *Excalibur_c25599_358*, genotyped on populations segregating for this locus, BP1-BP6. For YR_6A610: SNPs *GENE_4021_496* and *Tdurum_contig29607_413*, genotyped on populations BP4-BP8). Meta-analyses of the bi-parental population datasets relevant to each of the two loci found highly significant association with yellow rust resistance scores for both YR_2A010 (*P* < 0.001) and YR_6A610 (*P* < 0.001) (Supplementary Table S11; Supplementary Fig. S3). Accordingly, bi-parental analysis undertaken provided independent validation of both YR_2A010 and YR_6A610.

## Discussion

### Properties of the wheat association mapping panel

We assembled and genotyped an association mapping panel of 480 wheat cultivars, representing a valuable resource for dissecting the underpinning genetics of North-west European wheat germplasm developed across ~ 90 years of crop breeding. Our population was relatively large in comparison with other published wheat association mapping panels (e.g. the median population size of the 17 wheat panels used for GWAS cited in this manuscript is 273). Linkage disequilibrium in the WAGTAIL panel decayed at rates comparable to that typically observed in other inbred cereal crop species (e.g. Roncallo et al. 2021). While these rates are around an order of magnitude higher than that observed in outbreeding cereal crops such as maize (*Zea mays*) (e.g. 0.34 Mbp at a genome-wide level, Ertiro et al. 2020), the development of new cultivars via crossing means that association mapping panels consisting of collections of cultivars and breeding lines can be considered as pseudo-outbreeding populations that have been subjected to strong selection for beneficial alleles and allelic combinations (Rostoks et al. 2008). Thus, while lower genome-wide genetic marker numbers are required to identify genetic loci compared to an outbreeding crop like maize, the pseudo-outbreeding nature of the panel due to the crosses made by breeders results in elevation in genetic recombination levels of throughout much of the genome compared to purely inbreeding species.

Population substructure was evident in the panel, predominantly due to a combination of year of cultivar release, cultivar country of origin and spring/winter seasonal growth habit phenotype. Such substructure is a common feature of wheat association mapping panels (e.g. Bentley et al. 2014; Mellers et al. 2020; Walkowiak et al. 2020) and related cereal crops such as barley (e.g. Cockram et al. 2010), and is due to historic and/or recent similarities in the shared ancestry of the lines. If this is not accounted for, the frequency of false-positive associations can increase, due to causes other than close linkage between the genetic marker and QTL (Cockram and Mackay 2018). After statistical adjustment for substructure and kinship, plots of expected versus observed marker–trait significances for our disease traits indicated that the population stratification present in our panel was adequately accounted for. Power and precision to detect marker–trait associations in association mapping panels via GWAS relies on numerous factors, including population size and the amount of historic genetic recombination captured (Cockram and Mackay 2018). Estimation of the power of an association mapping panel to detect marker–trait associations provides a priori expectations of experimental design. While this is standard practice in human studies, it is not commonly applied in crops. Our power analyses indicated that the association mapping had relatively good power to detect loci, even when the percentage of the variation explained by a given locus was relatively low, indicating that association mapping panels of this size or greater are likely required for detection of quantitative sources of resistance in modern wheat cultivars.

### The genetic architecture of disease resistance in North-Western European wheat

Our analysis indicated that field resistance to the four target foliar diseases were under complex genetic control, with 34 replicated resistance loci identified across three of the four target diseases. Of the seven ‘multi-resistance’ genetic loci identified, six controlled resistance to two or all three of the target biotrophic diseases. A total of 87 permanently named loci have been identified for yellow rust (Rosewarne et al. 2013; Wang and Chen 2017; Catalogue of Gene Symbols of Wheat 2024) and 85 for brown rust (Koláriková et al. 2023; Catalogue of Gene Symbols of Wheat 2024), with many additional loci reported that have yet to be given formal *Yr* or *Lr* nomenclature. Resistance alleles at 70 named powdery mildew resistance genes (*Pm*) have been reported (Catalogue of Gene Symbols of Wheat 2024. See also review by Zou et al. 2023). Some wheat adult plant resistance genes confer resistance against two or more biotrophic pathogens, a characteristic that has been suggested to be an indicator of the durability of resistance. These include the following three loci, each conferring resistance to yellow rust, brown rust, stem rust and/or powdery mildew: *Yr18/Lr34/Pm38/ Sr67* (Spielmeyer et al. 2005; Lillemo et al. 2008), *Yr29/Lr46/Sr58/Pm39* (Lagudah 2011), *Yr30/Lr27/Sr2* (Mago et al. 2011) and *Yr46/Lr67/Sr55/Pm46* (Herrera-Foessel et al. 2014; Moore et al. 2015) (although we found no evidence for these presence of these loci in our European wheat panel). The ‘multi-resistance’ genetic loci identified here are defined as linked genetic loci rather than a single underlying gene. However, it may be possible that for some, the underlying gene may confer resistance to more than one disease. The most notable of our ‘multi-resistance’ loci, based on GWAS significance and number of trials identified in, included:

1. Yellow/brown rust locus YR_2A010/BR_2A015: Resistance at this locus on the short arm of wheat chromosome 2A was conferred by the *Ae. ventricosa* 2N^v^S introgression. Thirty-two per cent of the cultivars in our GWAS panel carried this introgression on chromosome 2A, as defined by our 162-SNP haplotype. This introgression is a well-known source of resistance to multiple diseases, including yellow rust (*Yr17*) (Fang et al. 2011), brown rust (*Lr37*) (Xu et al. 2018), stem rust (*Sr7a*, *Sr38*) (Turner et al. 2016), eyespot (Doussinault et al. 1983), wheat blast (Cruz et al. 2016; Wu et al. 2022) and cereal cyst nematode resistance (Jahier et al. 2008). While rust resistance conferred by *Yr17* and *Lr37* have been widely overcome (Bayles et al. 2000; UKCPVS, 2022), the 33 Mbp *Ae. ventricosa* segment is rich in NLR genes, with increased numbers of NLRs relative to the equivalent region in the wheat reference genome assembly (Gao et al. 2021). Indeed, our results indicate that 2N^v^S carries effective sources of yellow and brown rust resistance in addition to the previously overcome resistance genes *Yr17* and *Lr37*, agreeing with recent reports by Wang et al. (2023). This introgression has also been associated with increased grain yield (Gao et al. 2021; Juliana et al. 2019) and reduced lodging (Gao et al. 2021). Directional selection for 2N^v^S was evident in our panel, with notable change in frequency of the 2N^v^S haplotype in wheat pedigree over time: first introduced via ‘VPM1’ in the early 1980s (Dyck and Lukow 1988), it was passed onto several cultivars, including ‘Rendezvous’—a frequently used parent in the European pedigree (Fig. 7b) (Fradgley et al. 2019). ‘Rendezvous’ is a parent of subsequent parents that are frequently used in the pedigree, such as ‘Lynx’, ‘Hussar’ and ‘Tofrida’ (Fig. 7b, c) (Fradgley et al. 2019). Notably, while we found ‘Aardvark’ to lack 2N^v^S, it was a parent for seven cultivars which possess the introgression. Analysis of the pedigrees of these seven lines indicates that ‘Aardvark’ most likely carries 2N^v^S. Previous studies have noted genotypic discrepancies for ‘Aardvark’ (Corsi et al. 2020), indicating that either the incorrect germplasm was used here, or that residual heterozygosity was present within the cultivar when it was being used by breeding companies for crossing within the pedigree. The 2N^v^S introgression has previously been identified as a possible explanation for the very strong signals for directional selection in winter wheat across more than seventy years in the USA (Ayalew et al. 2020). Indeed, 48% of the most recent cultivars in our panel (from 2008 to 2010) contained the introgression. Similar strong selection is reported in wheat cultivars developed by CIMMYT in Mexico: frequency across all CIMMYT genotypes released between the 1990s to the early 2010s is ~ 24%, increasing to ~ 90% in lines released after 2015 (Gao et al. 2021; Juliana et al. 2019, 2020). Thus, the combination of multiple sources of disease resistance and beneficial yield traits may explain the continued strong selection for the 2N^v^S introgression in wheat breeding programmes over these periods. Rare putative (He et al. 2020; Xue et al. 2018) or observed (Wang et al. 2023) recombination between the 2N^v^S introgression and the native wheat chromosome 2A have previously been reported. However, we found no evidence for recombination within 2N^v^S in the 480 cultivars studied here. Thus, the two KASP genetic markers we developed (*Kukri_c18149_581* and *Excalibur_c25599_358*) are each capable of serving as a diagnostic tag for the extended putative 2N^v^S haplotype in our panel of cultivars, providing researchers and breeders with resources with which to track and manipulate this agronomically important genomic feature.
2. Yellow rust/brown rust locus YR_2B763/BR_2B777. Three named leaf rust resistance genes (*Lr50*, Brown-Guedira et al. 2003; *Lr58,* Kuraparthy et al. 2011; both originating from *T. timpoheevi; Lr82* from a wheat landrace, Bariana et al. 2022) and three named yellow rust resistance genes (*Yr5, Yr7* and *YrSP,* Marchal et al. 2018) are located on the long arm of chromosome 2B. Of these, physical map location based on anchoring to the wheat reference genome rules out all but *Lr50, Lr58* and *Lr82*—although it is currently unclear whether our locus represents resistance via all-stage or adult plant mechanisms. Accordingly, the chromosome 2B locus identified here may represent a novel yellow rust resistance gene in relatively close linkage to one or more brown rust resistance loci. Interestingly, a genetic locus controlling grain yield (*YLD_2B.4*, peak marker anchored on chromosome 2B at 766 Mbp) has recently been identified at this location in European wheat (White et al. 2021), indicating this genomic region may carry other beneficial alleles of agronomic relevance.
3. Brown rust/powdery mildew/yellow rust locus BR_4A714/PM_4A734/YR_4A738 (replicated in two, two and eight trials, respectively) located close to the telomere on the long arm of chromosome 4A. This region has recently been reported to confer resistance to both rust diseases (Liu et al. 2020; Kale et al. 2022) and to powdery mildew (Liang et al. 2022). Of the named resistance loci, the all-stage brown rust resistance gene *Lr28* effective against numerous *Pt* pathotypes (e.g. Bipinraj et al. 2011) is located in this region. *Lr28* is thought to have originated in wild wheat species, having been found in *Aegilops speltoides* (Naik et al. 1998), *Ae. crassa, Ae. juvenalis*, *Ae. triuncialis* and *T. timpoheevii* (Koláriková et al. 2023). The leaf rust resistance locus on the long arm of bread wheat chromosome 4A identified by Kale et al. (2022) in the European cultivar ‘Attraktion’ was noted to be in a genomic region shown to carry a 26-Mbp region of high sequence divergence with the wheat reference genome sequence (indicative of a chromosomal introgression from a wheat relative), and was identical by descent to an introgression carried in the UK cultivar ‘Robigus’. Indeed, via SNP array genotyping and analysis of pedigree records, this region in ‘Robigus’ has previously been reported to likely to originate from a wild wheat relative, potentially *T. dicoccoides* (Przewieslik-Allen et al. 2021). ‘Robigus’ is notable in its prominence in the UK wheat pedigree (Fradgley et al. 2019), highlighting the usefulness of alien chromosome introgression in European bread wheat resistance genetics, and the potential that *Lr28* may underlie the GWAS hit BR_4A714.
4. The yellow/brown rust locus YR_6A002/BR_6A016 on the short arm of chromosome 6A was replicated in five and three trials, respectively, and was validated in our study in bi-parental populations. This locus has recently been identified as a source of good yellow rust resistance at the adult plant stage in a UK wheat multi-founder population (*QYr.niab-6A.1*, based on peak SNP *BS00011010_51* on 6A at 19 Mbp, Bouvet et al. 2022c), further supporting the efficacy of this locus for rust resistance in the field.

### Additional genetic loci conferring strong resistance

In addition to the replicated GWAS hits that clustered into ‘multi-resistance’ loci, replicated genetic loci conferring resistance to single diseases were also identified. Notable amongst these were:

PM_1A003: Based on physical map location, PM_1A003 (chromosome 1A at ~ 3 Mbp) likely corresponds to the cloned powdery mildew resistance gene *Pm3* (Yahiaoui et al. 2004), located at 4.5 Mbp on chromosome 1A in the wheat reference genome. Previous work on a limited number of wheat cultivars indicated that of the ~ 10 known *Pm3* resistant alleles (*PM3a*-*Pm3j*), European wheat commonly carries *Pm3d* and *Pm3g* (Tommasini et al. 2006). Our findings that PM_1A004 confers field resistance to powdery mildew in both the UK and Denmark, combined with the recent finding that *Pm3a* was also the likely source of powdery mildew field resistance in a European multi-founder wheat population assessed in field trials in Germany (Stadlmeier et al. 2019), indicates that allelic variation at *Pm3* remains a good source of field resistance in European environments. Given *Pm3* alleles have been deployed in modern wheat cultivars for around 90 years (Hsam et al. 2003), characterisation of the *Pm3* alleles present in current wheat cultivars will help protect against breakdown in resistance, and could also help inform the use of parental lines carrying contrasting *Pm3* alleles for F_1_ hybrid varietal development.

YR_6A610: Based on its physical map location (chromosome 6A at ~ 610 Mbp) and its notably strong effect on resistance, YR_6A610 likely corresponds to a resistance locus recently identified in a European wheat multi-founder population (*QYr.niab-6A.3*, Bouvet et al. 2022c), as well as in smaller European GWAS panels grown in Europe (Germany and Austria; based on SNP *Tdurum_contig29607_413*, Shahinnia et al. 2022) and beyond (Norway, Austria, China; *QYr.nmbu.6A*, Lin et al. 2023). Further, we independently validated this locus via construction and analysis of bespoke bi-parental populations. Thus with trials spanning 2012–2021, these datasets (Bouvet et al. 2022c; Shahinnia et al. 2022; Lin et al. 2023, and the work we present here) collectively indicate that YR_6A610 has provided a strong source of yellow rust field resistance in European environments for at least ten years. Here we provide KASP genetic markers to track this locus for breeding and research purposes.

Of the five Septoria tritici blotch genetic loci identified, none were replicated. This reflects in some ways previous studies that find ST resistance to be controlled by numerous loci of small effect (Brown et al. 2015), and so reported effects of individual genetic loci may not be replicated between trials, years (e.g. Stadlmeier et al. 2019) or separate studies, even though varietal resistance is largely repeatable (e.g. Supplementary Fig. S1). The complexity of the wheat genetics is also compounded by interaction with the high levels of standing genetic variation present in *Zt* populations (McDonald et al. 2022). Of the unreplicated Septoria tritici blotch loci, ST_2B150 was located within 5 Mbp of the yellow rust resistance locus YR_2B155. Located close by is the hybrid necrosis gene *Necrosis 2* (*Ne2*, Hewitt et al. 2022)*,* an intracellular nucleotide-binding leucine-rich repeat (NLR) immune receptor—allelic variation within is allelic to both the leaf rust resistance gene *Lr17* (Hewitt et al. 2022) and the yellow rust resistance gene *Yr27* (Athiyannan et al. 2022) and whose equivalent gene in the wheat reference genome is located at 157.7 Mbp (*TraesCS2B02G182800*). Depending on allele and genetic background, deletions of portions of the *Ne2* gene result in loss of disease resistance while retaining a necrotic phenotype (Hewitt et al. 2022), highlighting possible links between biotrophic and necrotrophic disease response.

### Breeding utility of loci identified in this study

The utility of the identified loci for disease resistance breeding is partly determined by their effect sizes, by the number, geographic breadth and temporal range of trials in which they were found to be significant, and by whether they have been successfully validated. However, the distribution of the alleles in the panel is also of considerable importance. If the resistance allele is very common, then investing in a marker-based selection strategy is less likely to be beneficial to a breeder, especially if the rare susceptible alleles are largely found in the older material in the panel (as we found here for yellow rust). On the other hand, if the resistant allele is rare, it is likely to be more useful for future breeding efforts, all other factors being equal. The distribution of alleles across the frequency spectrum is shown in Table 3. Across diseases, there are similar proportions of rare (resistance allele frequency ≤ 10%) and common (resistance allele frequency > 90%) resistance alleles, 15/96 and 16/96, respectively. Similarly, there are approximately equal proportions of moderately common (33/96) and moderately rare (32/96) resistance alleles in the association mapping panel (Table 3). Many of the rarest alleles (< 10% frequency) were only detected in one or two trials, possibly due in part to their rarity making detection harder. Of these, the replicated resistance loci that contribute to ‘multi-resistance’ locus MT25Mb-6 were of particular note. This included PM_4A734 (resistance allele frequency 6%), one of only two replicated powdery mildew resistance QTL identified, and BR_4A714 (resistance allele frequency = 3%), one of the most highly significant brown rust resistance genetic loci identified and introduced first into the pedigree via cv. ‘Robigus’ in 2002. These examples highlight the potential of exploiting currently rare disease resistance alleles for forward selection in breeding programmes.

**Table 3:** Grouping the 96 quantitative trait loci (QTL) identified by genome-wide association study (GWAS) by the frequency of resistance alleles in the association mapping panel

Disease	Frequency of resistance allele			
	0–10%	11–50%	51–90%	91–100%
BR	4	13	14	3
PM	4	5	2	3
ST	0	3	1	1
YR	7	11	16	9
Total	15	32	33	16

Brown rust (BR), powdery mildew (PM), Septoria tritici blotch (ST) and yellow rust (YR)

### Concluding remarks

Here we define numerous quantitative sources of disease resistance within elite wheat germplasm released over a 90-year period, finding chromosomal regions conferring resistance to more than one disease, as well as highlighting the role of chromosomal introgressions from wild wheat relatives in the resistance profiles of modern wheat. Notably, the first incursions of genetically diverse *Pst* isolates that swept across the European agricultural landscape from 2011 (e.g. Hubbard et al. 2015; Hovmøller et al. 2016; UKCPVS 2022) resulting in rapid changes in YR resistance due to break down of previously effective durable sources of resistance, were beginning to occur across the duration of our YR field trials. Thus, our YR results catalogue the effective sources of resistance to these new endemic *Pst* races. Finally, none of the three adult plant rust resistance genes cloned to date, *Yr18/Lr34/Sr67/Pm38, Yr36* (Fu et al. 2009) and *Yr46/Lr67/Sr55/Pm46* (estimated here as being located on the wheat reference genome on chromosome 7D:474 Mbp, 6B:136 Mbp and 4D:405 Mbp, respectively), were identified as sources of resistance in our panel. If they are indeed absent, this may be due to their origin from unadapted germplasm (*Yr18* and *Yr46* originated from Chinese landraces and central American wheat, respectively) (Krattinger et al. 2009; Singh et al. 1998) or different wheat species (*Yr36*), and suggests biotrophic fungal pathogen resistance could be rapidly enhanced in the European genepool via use of these loci. Collectively, the information generated here will help optimise sources of genetic resistance present in elite wheat, so providing a baseline from which new resistance loci can be introduced.

## Supplementary Information

Below is the link to the electronic supplementary material.

Supplementary file1 (DOCX 279 KB)

Supplementary file2 (DOCX 8784 KB)

Supplementary file3 (DOCX 171 KB)

Supplementary file4 (XLSX 45931 KB)

Supplementary file5 (DOCX 14 KB)

## Data Availability

All datasets used are either included as supplementary materials, or are publicly available.
