# Supplementary material for: Genome-wide association analysis identifies seven loci conferring resistance to multiple wheat foliar diseases, including brown and yellow rust resistance originating from Aegilops ventricosa
Source: Theor Appl Genet. 2025 Jun 2;138(6):133. doi: 10.1007/s00122-025-04907-x (PMC12129864; doi:10.1007/s00122-025-04907-x)
Supplement: Supplementary file 1 — Supplementary file1 (DOCX 279 KB) [file 122_2025_4907_MOESM1_ESM.docx]

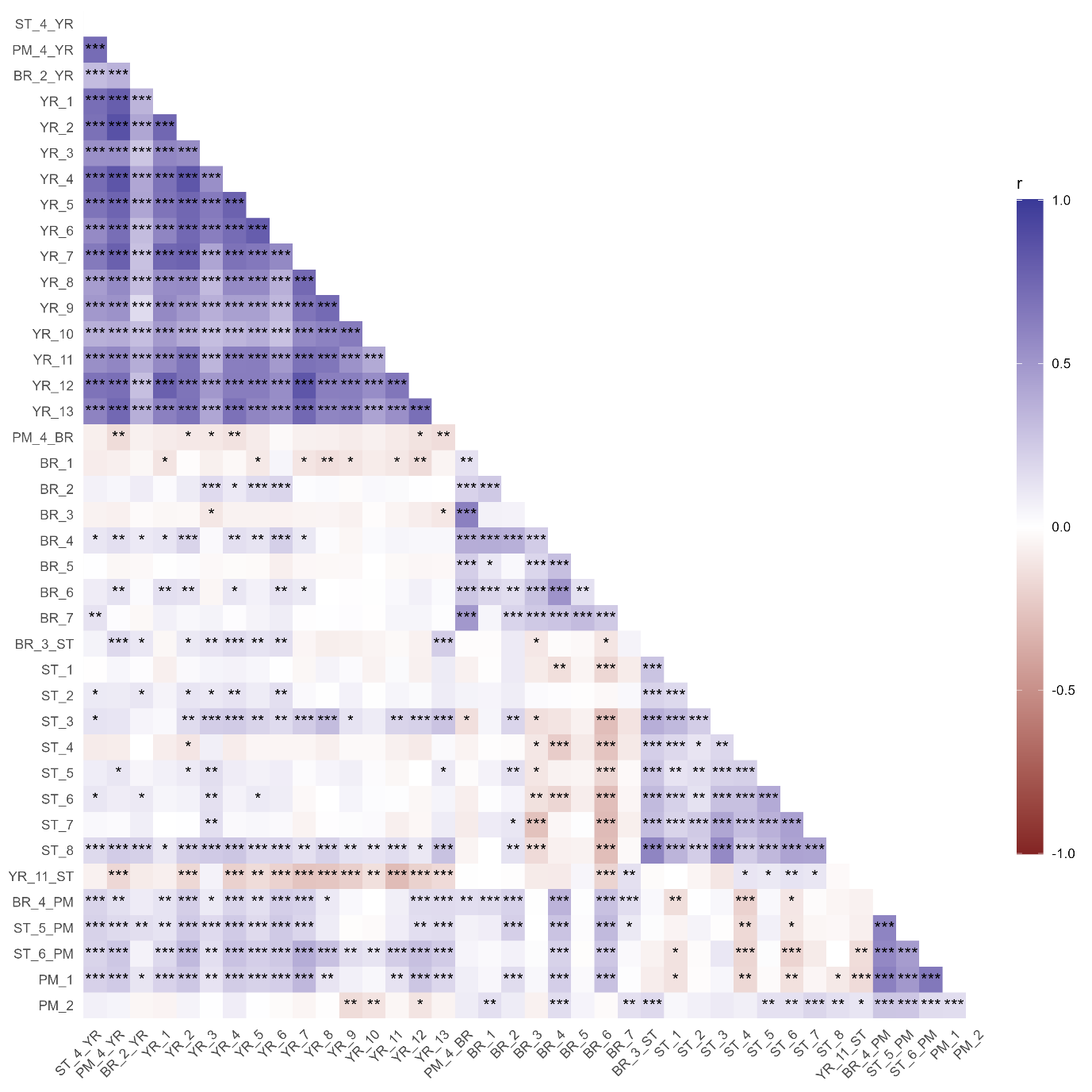


**Supplementary Figure S1.** Correlation of percentage disease infection scores in the WAGTAIL wheat association mapping panel between all traits and trials, using the variety means on untransformed data. Trial codes: BR = brown rust, PM = powdery mildew, ST = Septoria tritici blotch, YR = yellow rust. Significance: P = 0.05^*^, P = 0.01^**^, P = 0.001^***^. Where more than one disease score date was recorded for a given trial, here data from the last date was used for correlation analysis.
