# Supplementary material for: Genome-wide association analysis identifies seven loci conferring resistance to multiple wheat foliar diseases, including brown and yellow rust resistance originating from Aegilops ventricosa
Source: Theor Appl Genet. 2025 Jun 2;138(6):133. doi: 10.1007/s00122-025-04907-x (PMC12129864; doi:10.1007/s00122-025-04907-x)
Supplement: Supplementary file 2 — Supplementary file2 (DOCX 8784 KB) [file 122_2025_4907_MOESM2_ESM.docx]

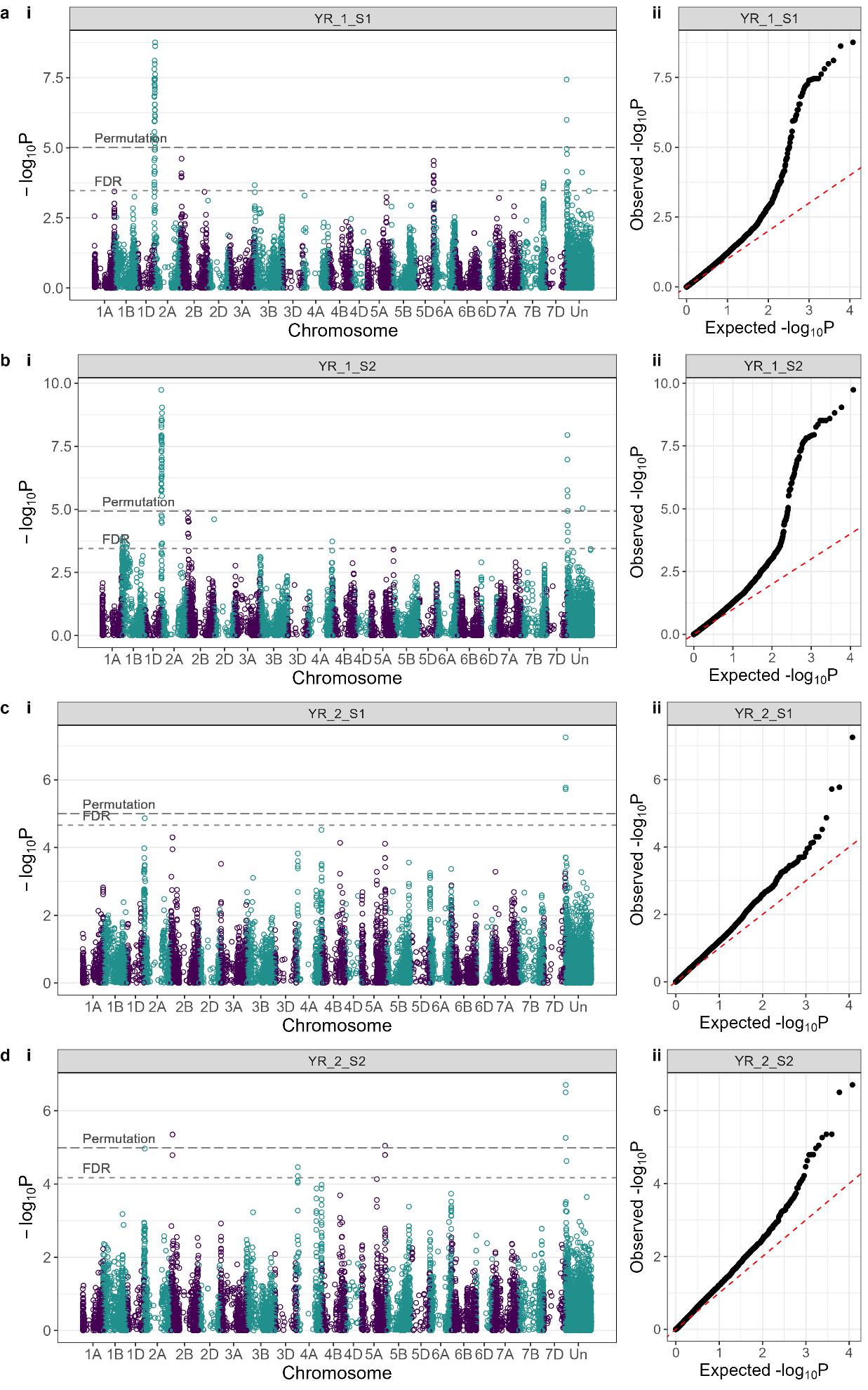


**
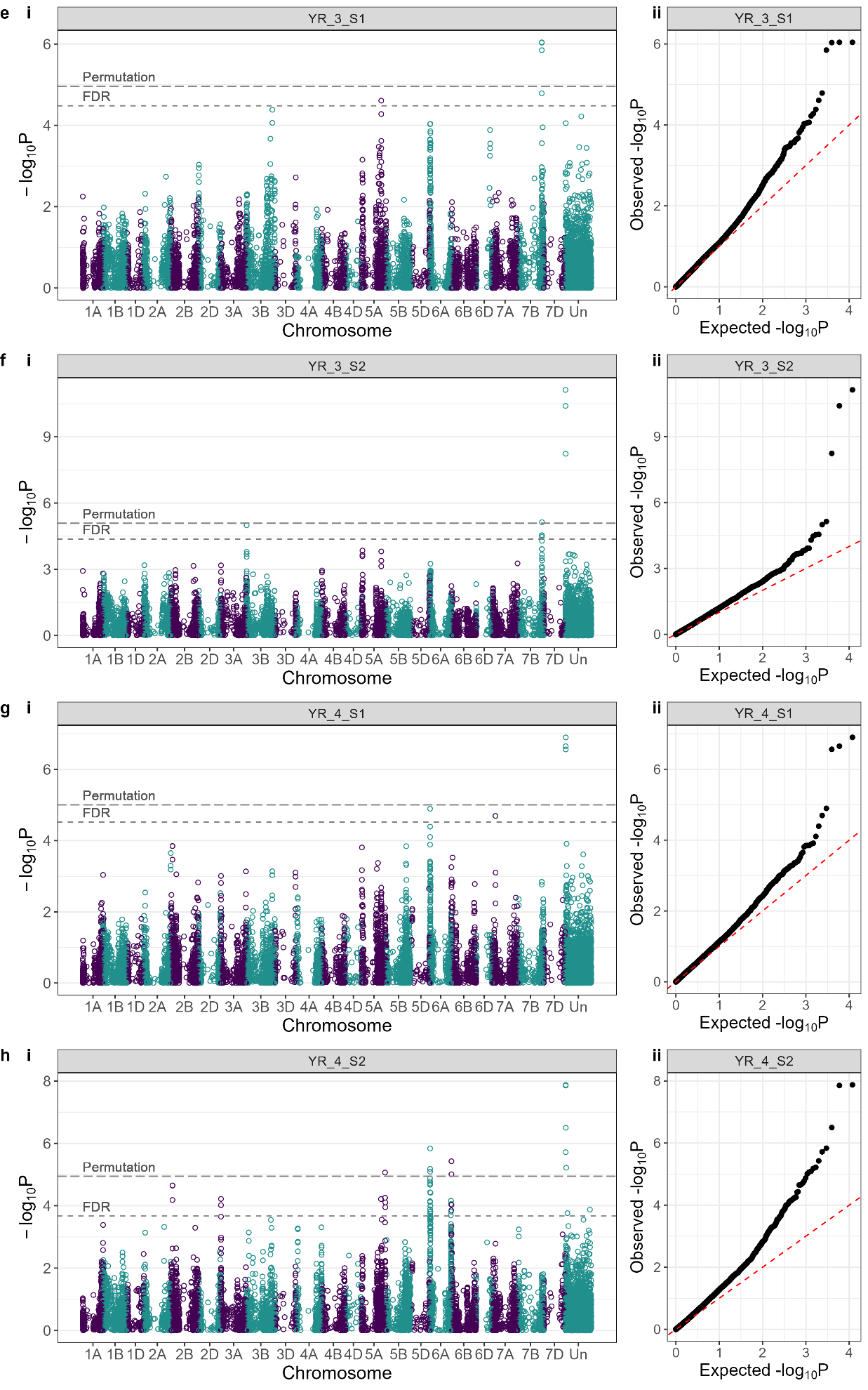
**

**
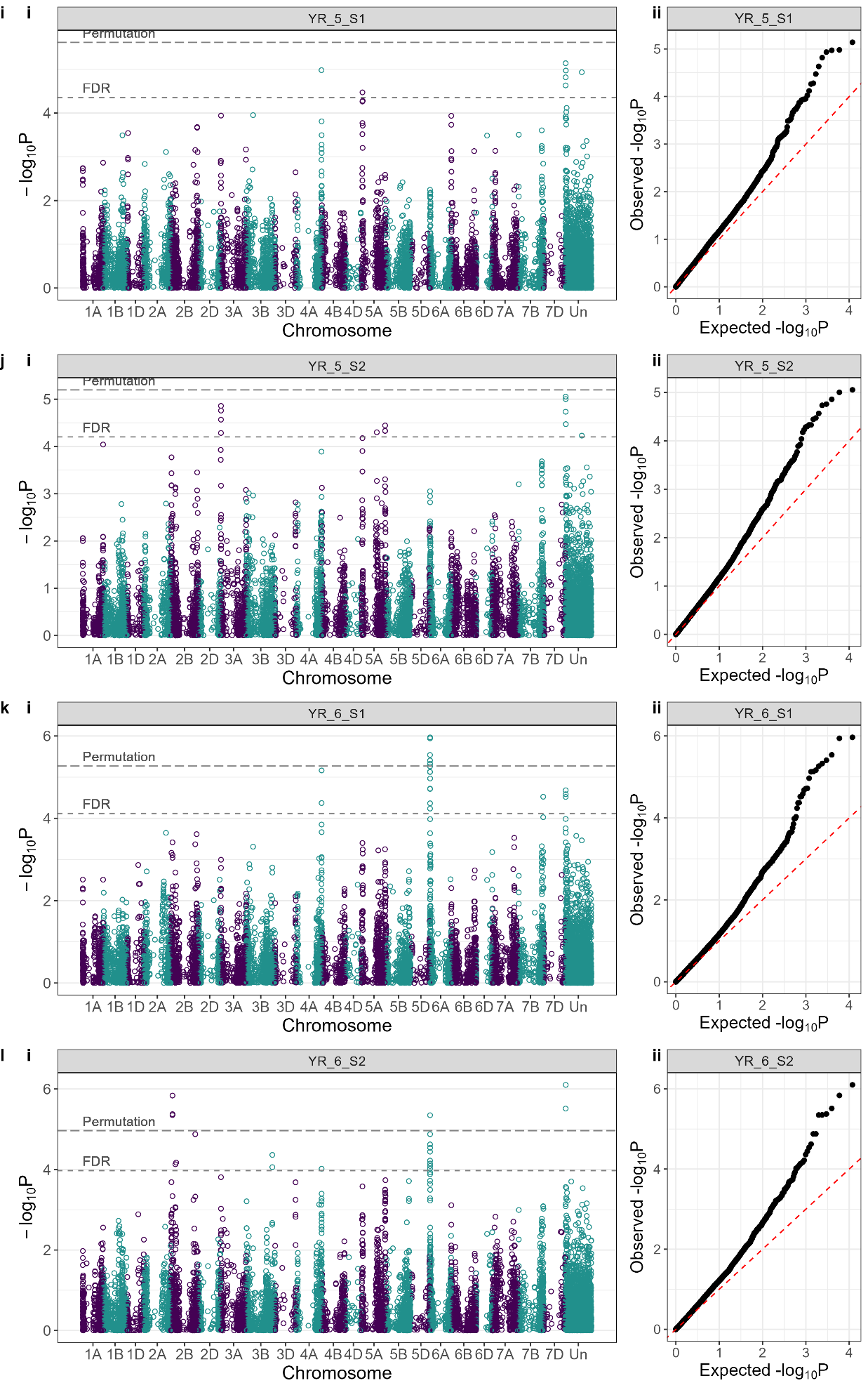
**

**
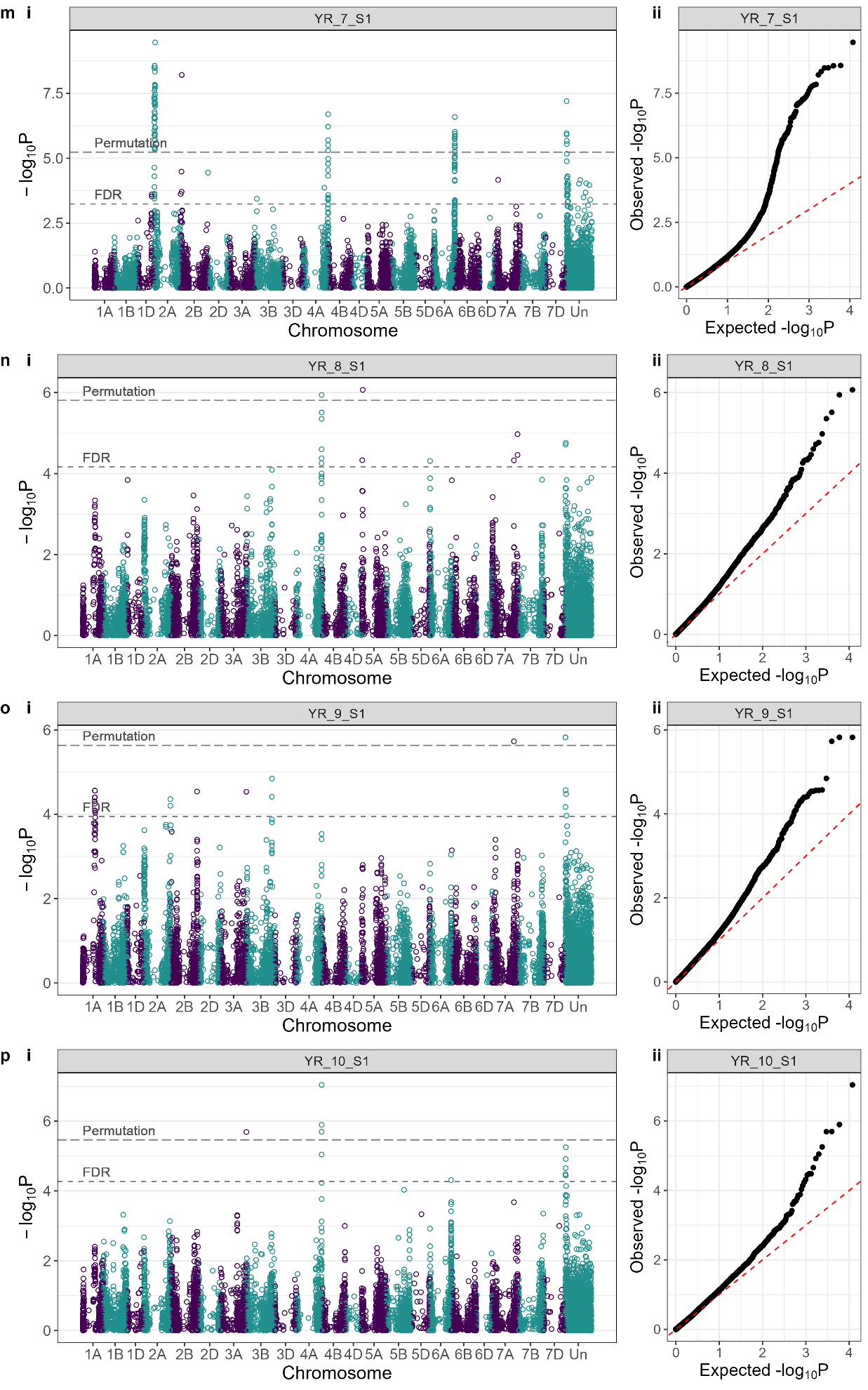
**

**
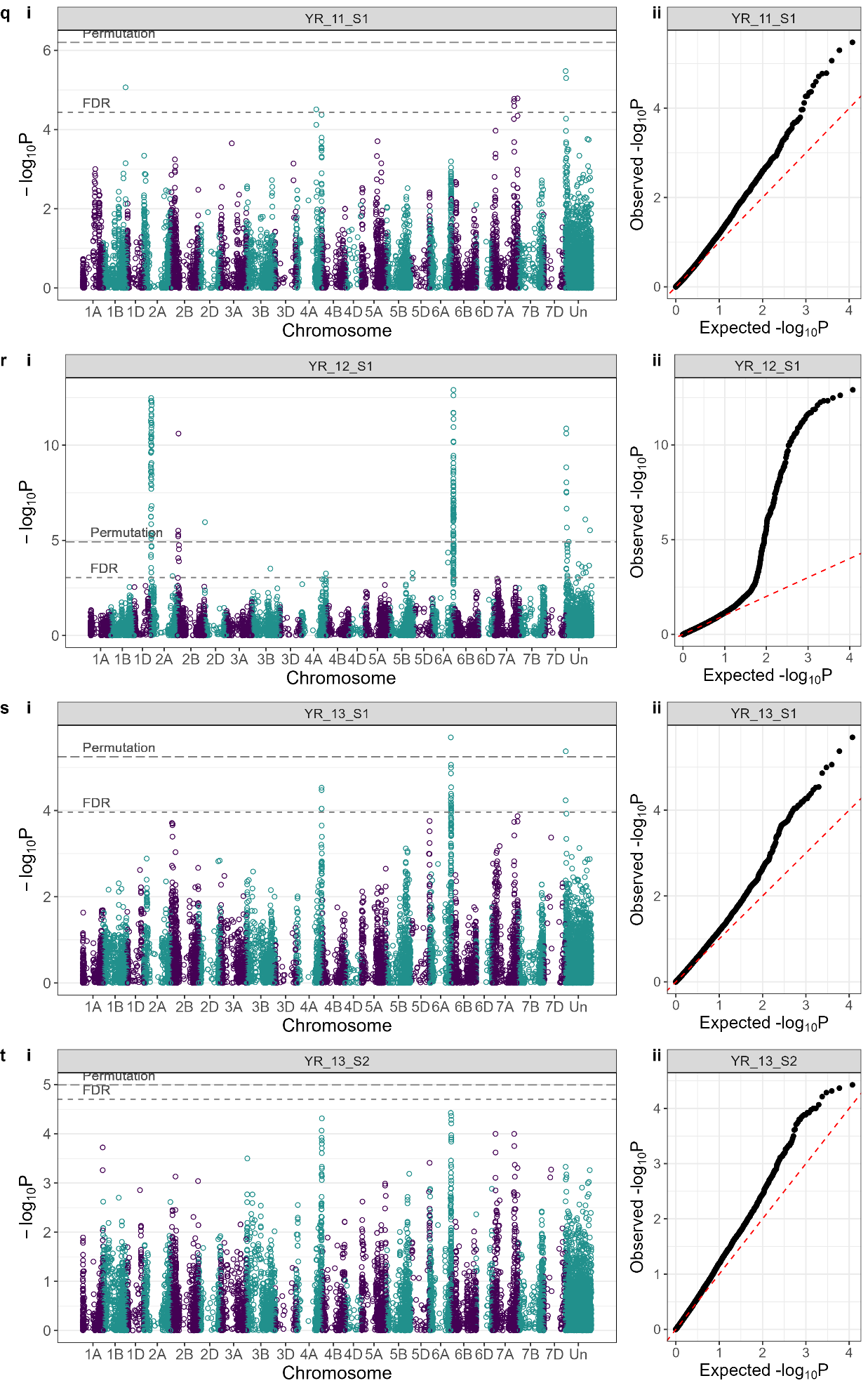
**

**
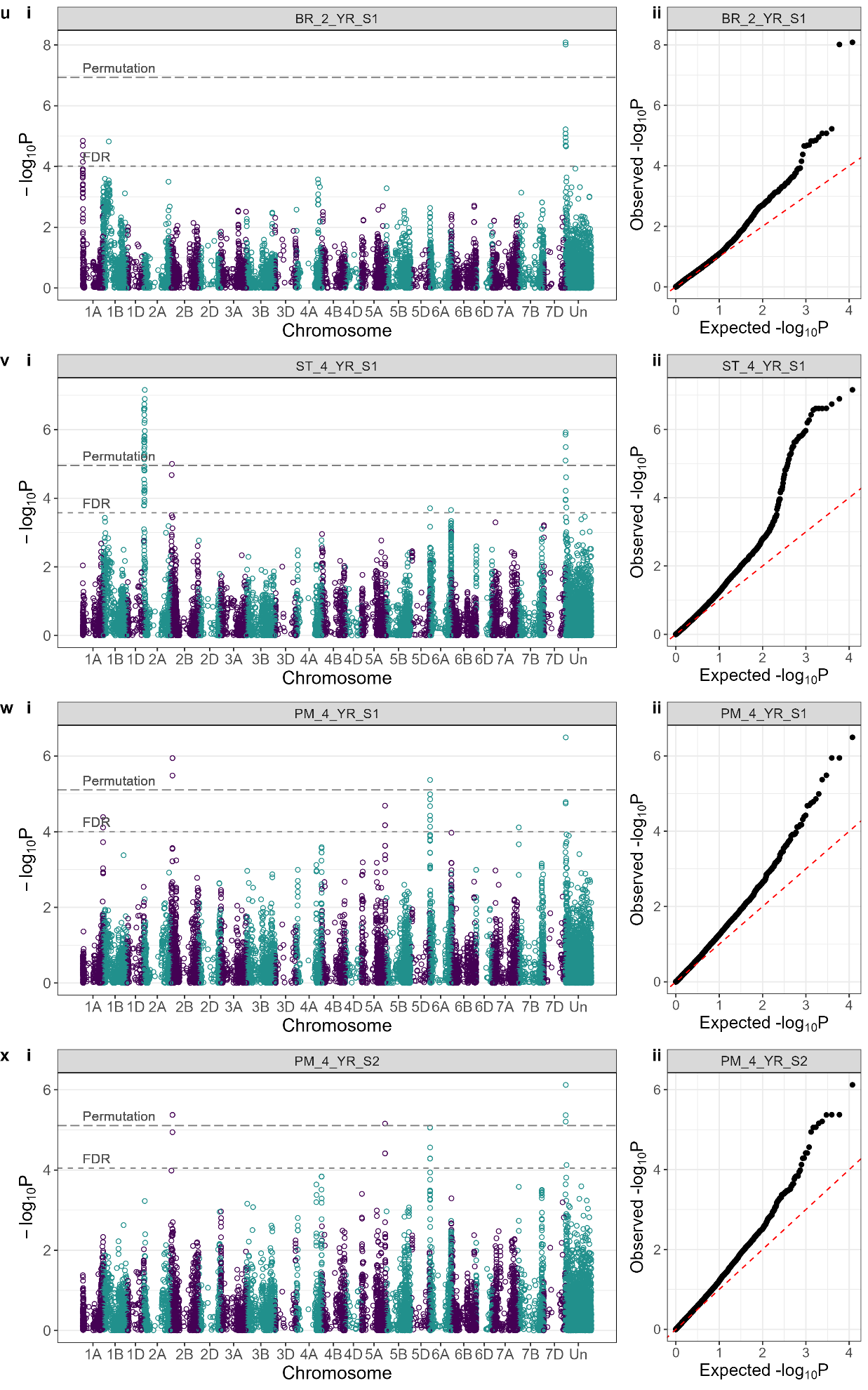
**

**
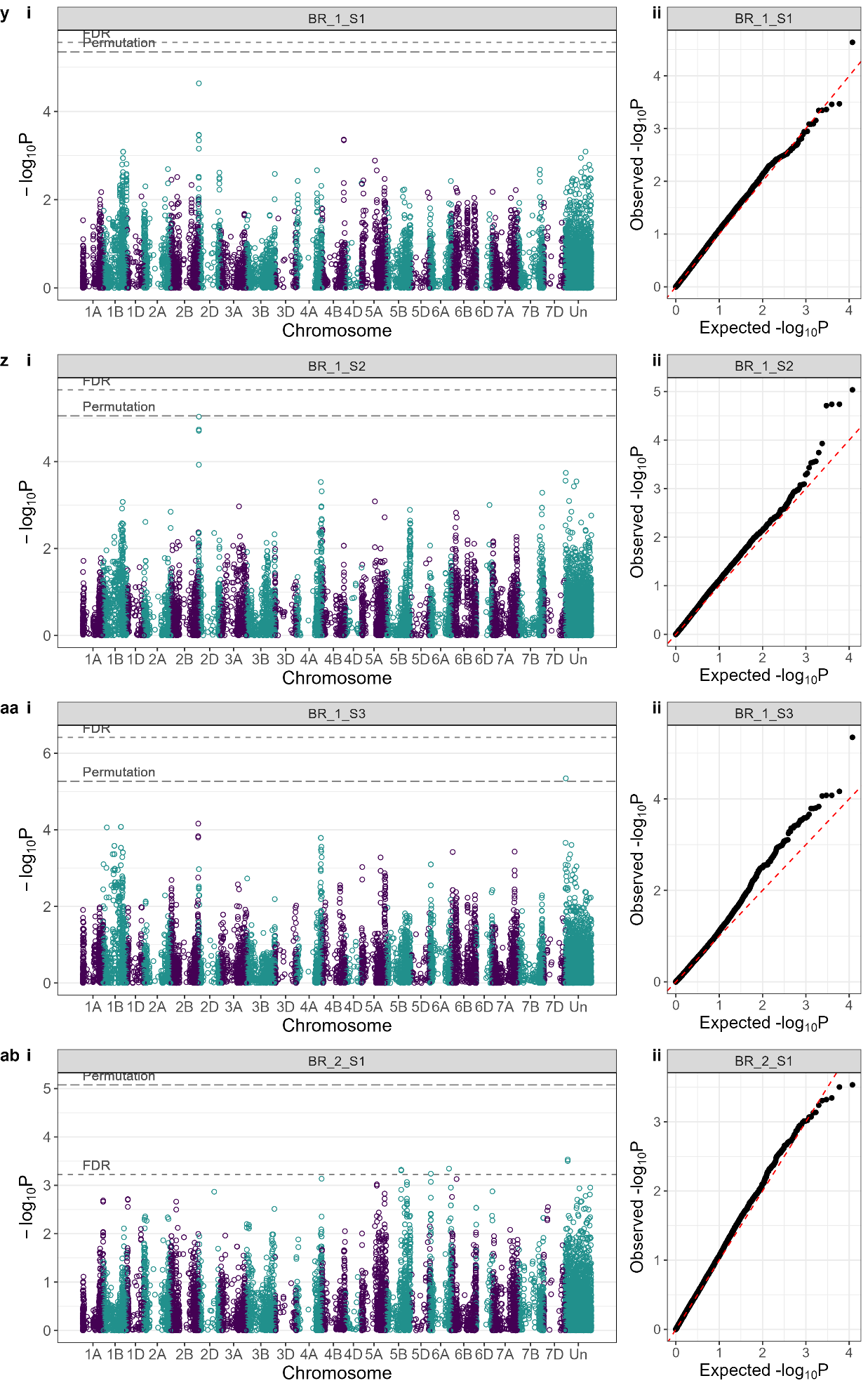
**

**
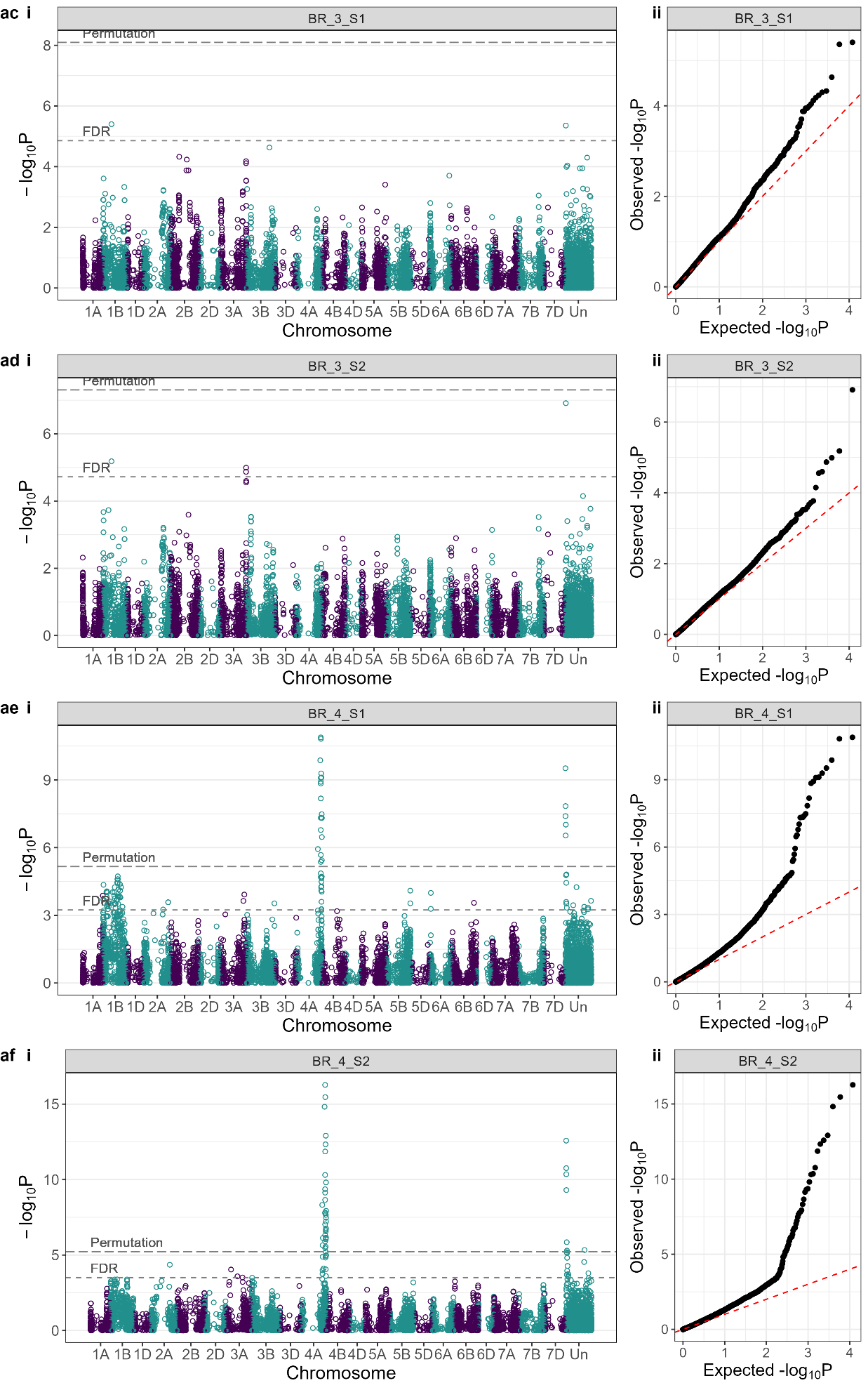
**

**
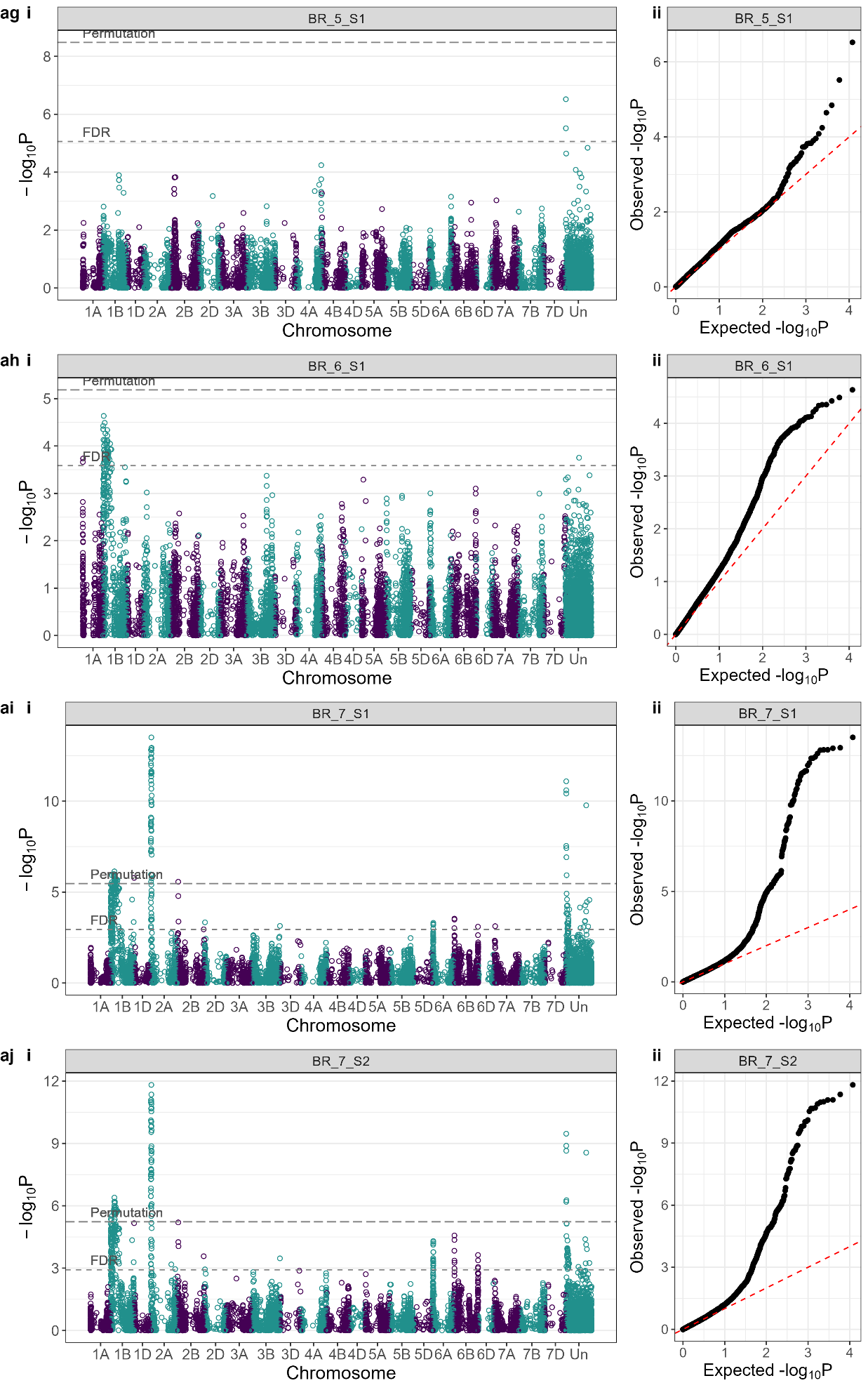
**

**
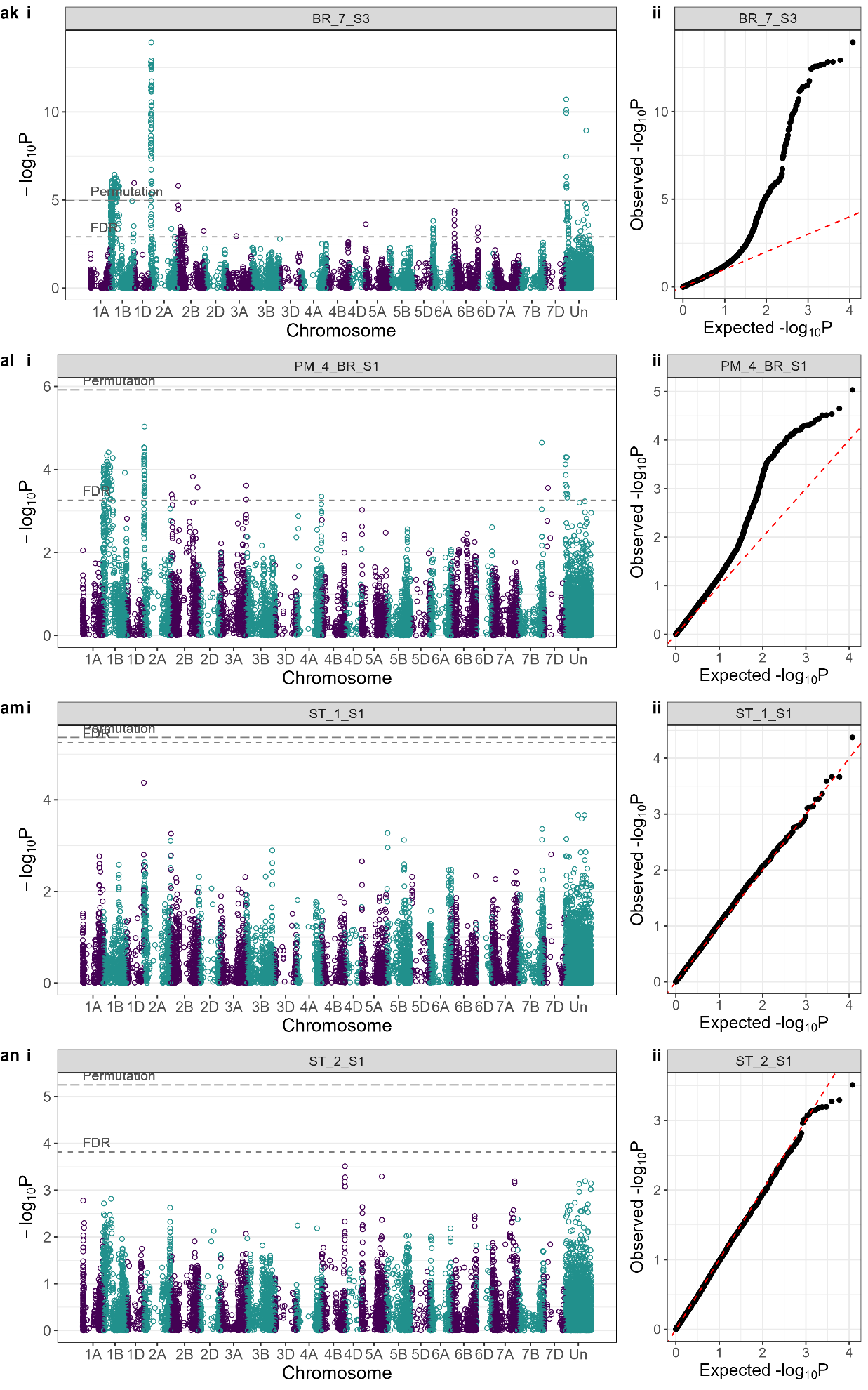
**

**
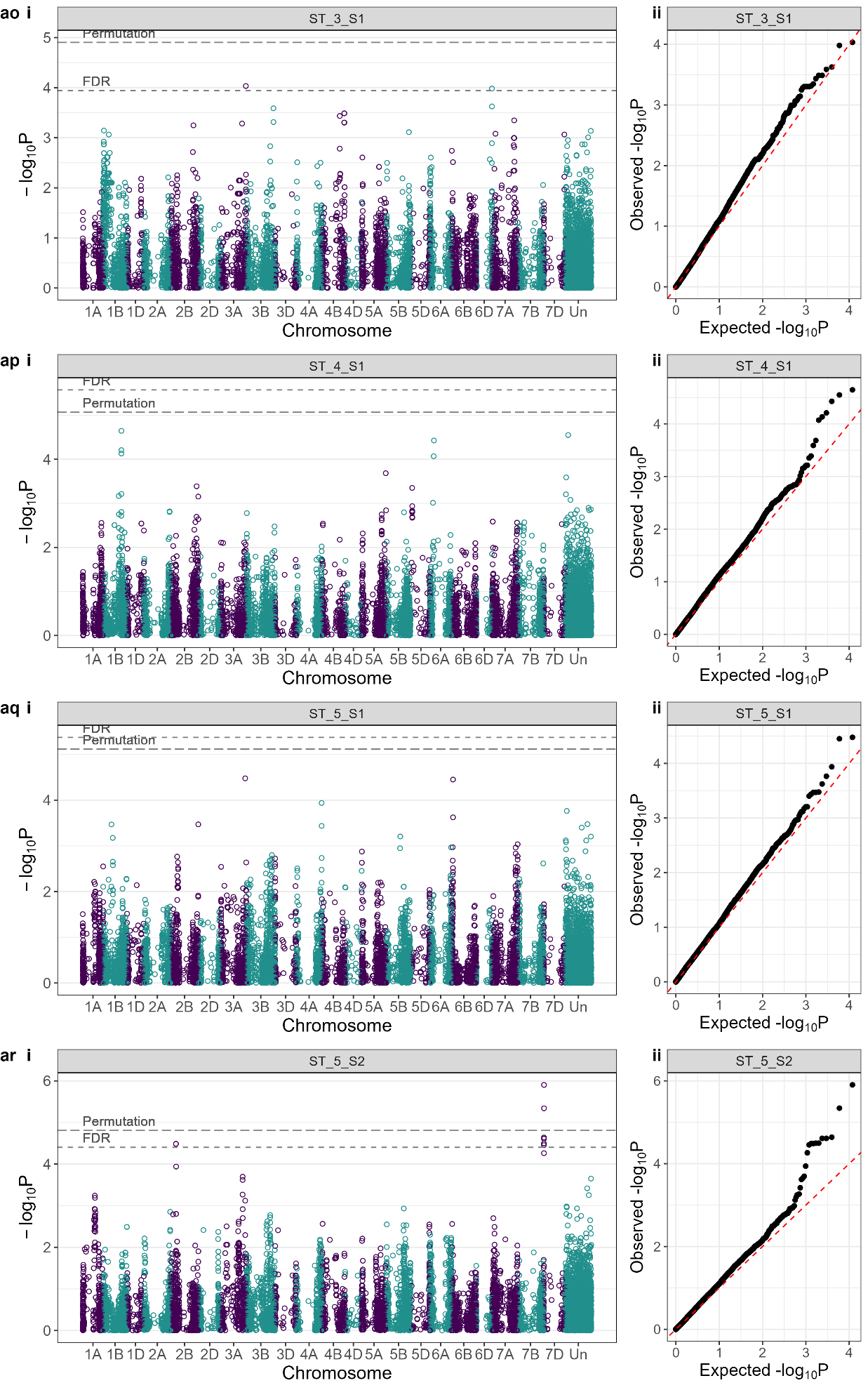
**

**
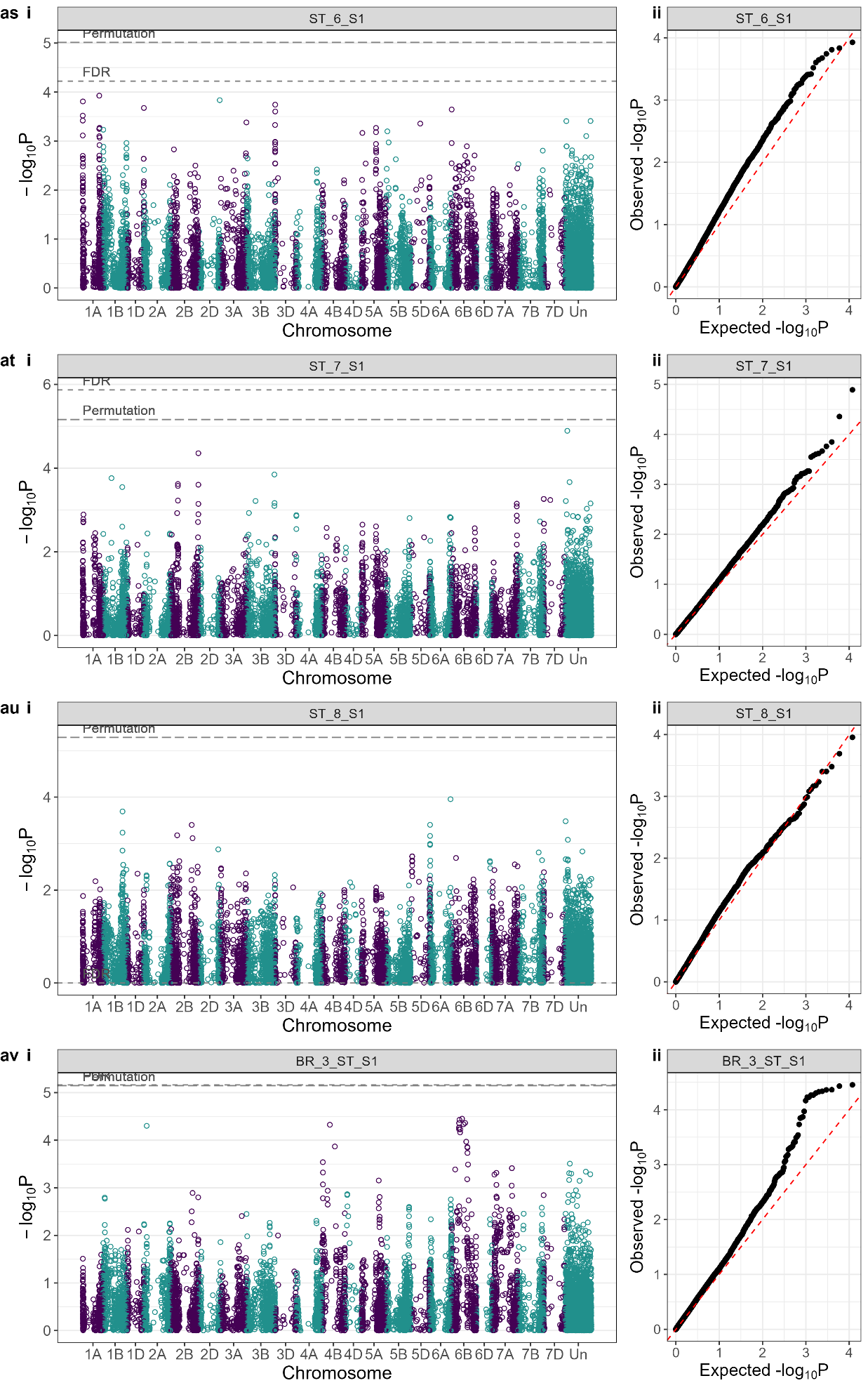
**

**
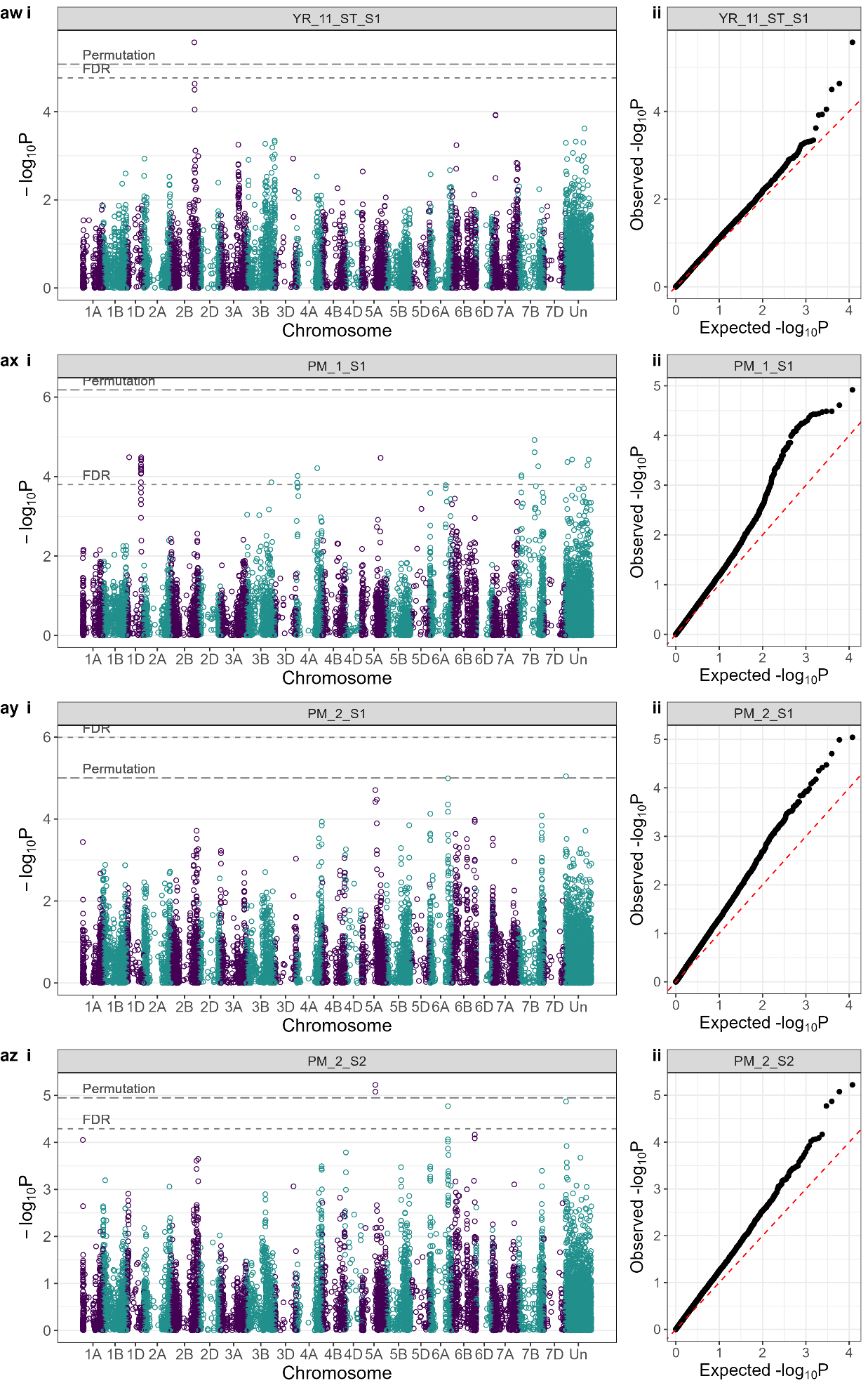
**

**
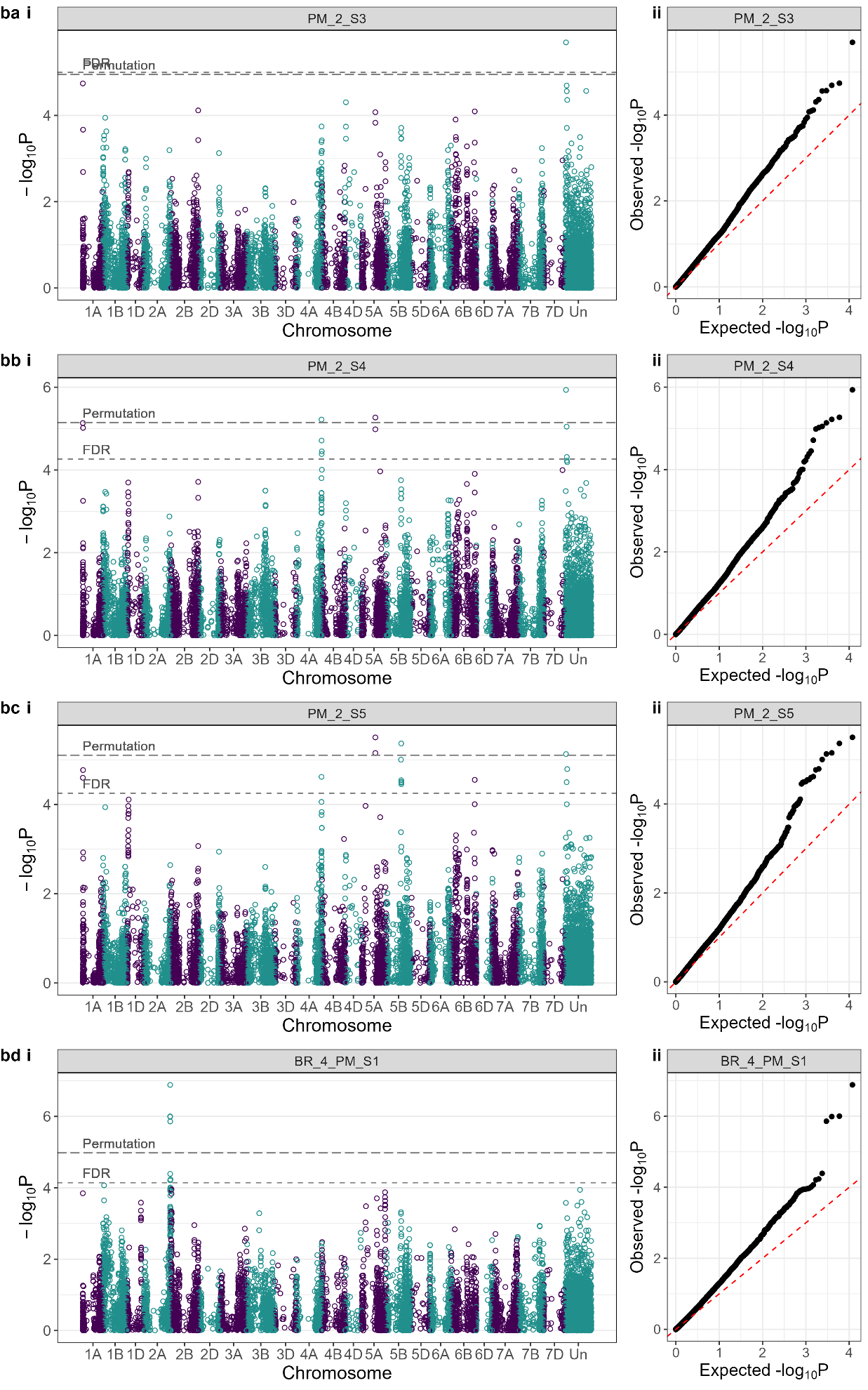
**

**
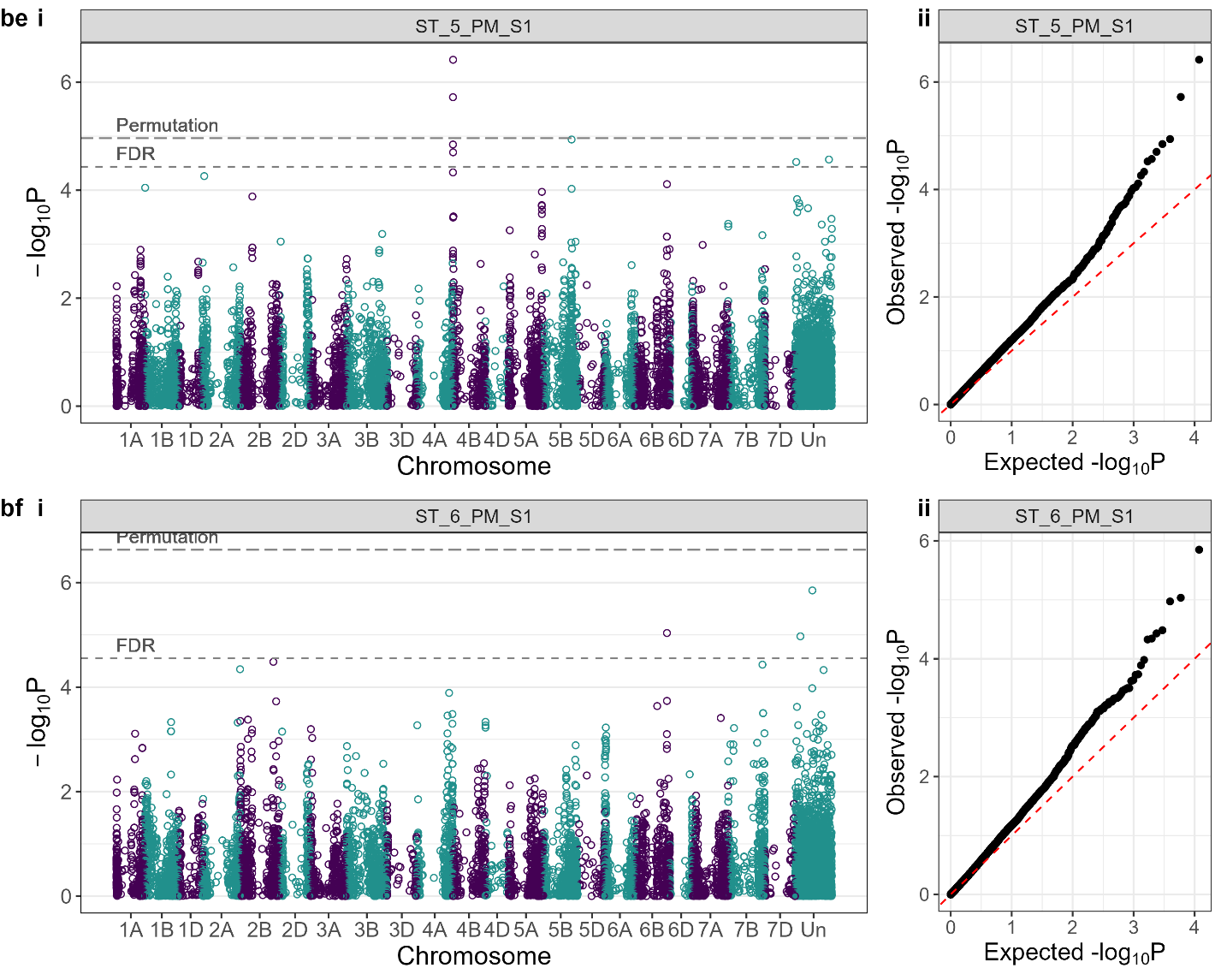
**

**Supplementary Fig. S2** Genome-wide association study (GWAS) results for all trial, disease, disease score timepoints analysed in the WAGTAIL bread wheat association mapping panel. BR = brown rust. PM = powdery mildew, ST = Septoria tritici blotch, YR = yellow rust. S1 = disease score timepoint-1, S2 = disease score timepoint-2. (i) Manhattan plots, (ii) Quantile-Quantile plots. FDR = false discovery rate significance threshold with *q*-value cut-off of *q* = 0.05. Permutation = permutated significance threshold with 1,000 permutations and *α* = 0.05.
