# Supplementary material for: Genome-wide association analysis identifies seven loci conferring resistance to multiple wheat foliar diseases, including brown and yellow rust resistance originating from Aegilops ventricosa
Source: Theor Appl Genet. 2025 Jun 2;138(6):133. doi: 10.1007/s00122-025-04907-x (PMC12129864; doi:10.1007/s00122-025-04907-x)
Supplement: Supplementary file 3 — Supplementary file3 (DOCX 171 KB) [file 122_2025_4907_MOESM3_ESM.docx]

**
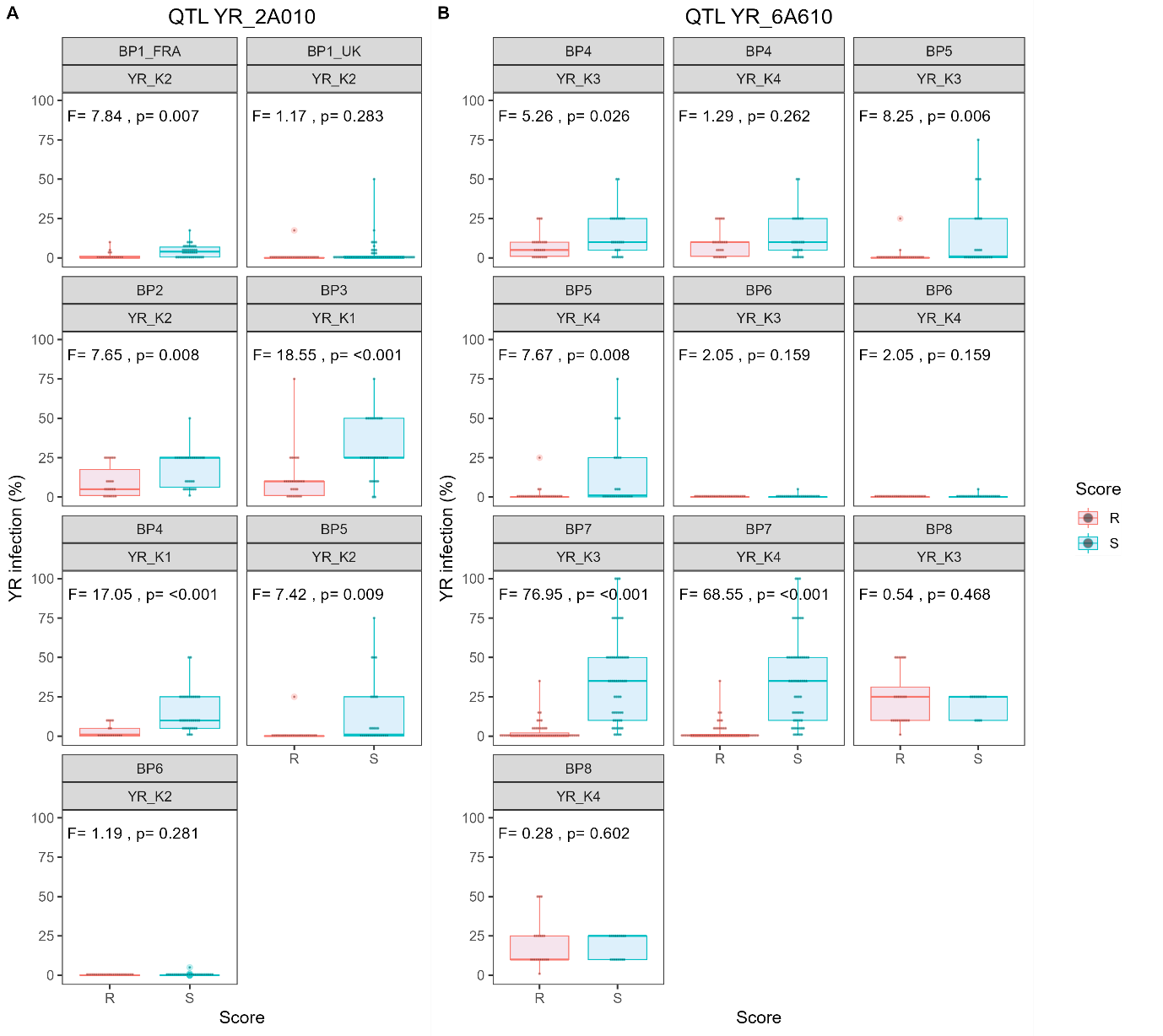
**

**Supplementary Figure S3**. Boxplots showing association between genetic marker genotype and yellow rust adult plant resistance in a series of eight bi-parental validation populations constructed to segregate for rust resistance quantitative trait loci (QTL) YR_2A010 (Panel A) and YR_6A610 (Panel B). Two KASP genetic markers were tested for each genetic locus: YR_K1 (90k SNP array marker *Kukri_c18149_581*) and YR_K2 (*Excalibur_c25599_358*) for locus YR_2A010, and YR_K3 (*GENE_4021_496*) and YR_K4 (*Tdurum_contig29607_413*) for locus YR_6A610. Bi-parental populations BP1-BP3 were predicted to segregate for YR_2A010. Bi-parental populations BP7 and BP8 were predicted to segregate for YR_6A610. Bi-parental populations BP4-BP6 were predicted to segregate for both YR_2A010 and YR_6A610. BP1 was grown in two locations: the UK (BP1_UK) and France (BP1_FRA). For each population/KASP marker combination presented, boxplots of yellow rust resistance (percentage infection) for the population lines that grouped into the resistant (R) and susceptible (S) KASP allele classes are shown. For boxplots, the line within the box represents the median, the bottom and top of boxes represent upper and lower quartiles, and lines below and above the boxes represent minimum and maximum values, respectively. One way ANOVA statistics shown on each panel: F values, with a larger F value indicating the variation is more likely caused by the susceptibility score than by chance, and the P-values < 0.05 indicate significant difference between resistant versus susceptible genotypic classes. Note, for YR_2A010 as two KASP markers YR_K1 and YR_K2 resulted in identical genotypic calls in the 90k SNP dataset, but on conversion to the KASP genotyping platform marker YR_K1 performed slightly more robustly than YR_K2, in those populations that both markers were genotyped, here we show results for YR_K1 only.
