# Supplementary material for: Genome-wide association analysis identifies seven loci conferring resistance to multiple wheat foliar diseases, including brown and yellow rust resistance originating from Aegilops ventricosa
Source: Theor Appl Genet. 2025 Jun 2;138(6):133. doi: 10.1007/s00122-025-04907-x (PMC12129864; doi:10.1007/s00122-025-04907-x)
Supplement: Supplementary file 5 — Supplementary file5 (DOCX 14 KB) [file 122_2025_4907_MOESM5_ESM.docx]

**Supplementary Text 1**

Genotyping the panel with a 90,000 feature SNP array resulted in 26,015 polymorphic genetic markers. Of these, 877 represented markers where off-target variation was observed for a particular cluster of individuals in addition to the anticipated clustering. For example, a group of individuals had a null call presumably due to a DNA mutation affecting the assay (such as a deletion). These markers were manually curated and split to form two markers to capture off-target variations; for these, markers names were suffixed by an ‘a’ or ‘b’, which accounted for 1,729 of the 26,015 markers. After further the further processing described in the Methods, the final set of 11,858 markers used for GWAS included 976 of these suffixed markers.
